# The role of endothelial MERTK during the inflammatory response in lungs

**DOI:** 10.1371/journal.pone.0225051

**Published:** 2019-12-05

**Authors:** Yitong Li, Erika S. Wittchen, Elizabeth Monaghan-Benson, Cornelia Hahn, H. Shelton Earp, Claire M. Doerschuk, Keith Burridge

**Affiliations:** 1 Department of Cell Biology and Physiology, University of North Carolina at Chapel Hill, Chapel Hill, North Carolina, United States of America; 2 Marsico Lung Institute, University of North Carolina at Chapel Hill, Chapel Hill, North Carolina, United States of America; 3 Division of Pulmonary Diseases and Critical Care Medicine, Department of Medicine, University of North Carolina at Chapel Hill, Chapel Hill, North Carolina, United States of America; 4 Lineberger Comprehensive Cancer Center, University of North Carolina at Chapel Hill, Chapel Hill, North Carolina, United States of America; Emory University School of Medicine, UNITED STATES

## Abstract

As a key homeostasis regulator in mammals, the MERTK receptor tyrosine kinase is crucial for efferocytosis, a process that requires remodeling of the cell membrane and adjacent actin cytoskeleton. Membrane and cytoskeletal reorganization also occur in endothelial cells during inflammation, particularly during neutrophil transendothelial migration (TEM) and during changes in permeability. However, MERTK’s function in endothelial cells remains unclear. This study evaluated the contribution of endothelial MERTK to neutrophil TEM and endothelial barrier function. *In vitro* experiments using primary human pulmonary microvascular endothelial cells found that neutrophil TEM across the endothelial monolayers was enhanced when MERTK expression in endothelial cells was reduced by siRNA knockdown. Examination of endothelial barrier function revealed increased passage of dextran across the MERTK-depleted monolayers, suggesting that MERTK helps maintain endothelial barrier function. MERTK knockdown also altered adherens junction structure, decreased junction protein levels, and reduced basal Rac1 activity in endothelial cells, providing potential mechanisms of how MERTK regulates endothelial barrier function. To study MERTK’s function *in vivo*, inflammation in the lungs of global *Mertk*^*-/-*^ mice was examined during acute pneumonia. In response to *P*. *aeruginosa*, more neutrophils were recruited to the lungs of *Mertk*^*-/-*^ than wildtype mice. Vascular leakage of Evans blue dye into the lung tissue was also greater in *Mertk*^*-/-*^ mice. To analyze endothelial MERTK’s involvement in these processes, we generated inducible endothelial cell-specific (iEC) *Mertk*^*-/-*^ mice. When similarly challenged with *P*. *aeruginosa*, iEC *Mertk*^*-/-*^ mice demonstrated no difference in neutrophil TEM into the inflamed lungs or in vascular permeability compared to control mice. These results suggest that deletion of MERTK in human pulmonary microvascular endothelial cells *in vitro* and in all cells *in vivo* aggravates the inflammatory response. However, selective MERTK deletion in endothelial cells *in vivo* failed to replicate this response.

## Introduction

Expressed in many different tissues, the Mer receptor tyrosine kinase (MERTK) plays important roles during developmental, physiological, and pathological processes [1–6]. MERTK belongs to the TYRO3/AXL/MERTK (TAM) receptor tyrosine kinase family. All three TAM kinases are expressed in many adult tissues such as the nervous system, reproductive organs, the lungs, hematopoietic cells, the vasculature, and more [5–7]. This widespread expression pattern correlates with their involvement in various key cellular processes, including growth and differentiation, adhesion, migration, and immune regulation [6–9]. Among these, their importance in maintaining tissue homeostasis has been underscored by studies with single, double, and triple TAM kinase knockout mice [10–12]. Mice lacking MERTK develop mild autoimmunity [12, 13] while mice lacking all three TAM kinases develop severe autoimmunity, partially caused by hyperactivation of antigen-presenting cells and hyperproliferation of lymphocytes [14]. All three TAM kinases facilitate binding of apoptotic cells by phagocytic cells via their ligands [6, 15, 16]. MERTK kinase activity is crucial for the engulfment of apoptotic thymocytes by macrophages [17, 18]. The mechanism appears to be through MERTK-mediated activation of PKC and Rho GTPase signaling, which induces membrane and cytoskeleton reorganization in phagocytic cells, driving apoptotic cell uptake in a process known as efferocytosis [19, 20]. Inability to clear apoptotic cells due to the lack of MERTK can lead to retinitis pigmentosa [9].

Similar highly regulated cell-cell interactions and cytoskeleton remodeling seen during phagocytosis are also observed during leukocyte transendothelial migration (TEM) [21, 22]. TEM is an important process during the inflammatory response whereby leukocytes exit the circulation, cross the vascular endothelium, and migrate towards the site of injury or infection. This process is normally under strict control, as the endothelial barrier allows leukocyte emigration only upon proper stimulation. TEM facilitates immune surveillance and response, but when dysregulated, it can also contribute to inflammatory disorders such as atherosclerosis and acute respiratory distress syndrome. Studies on this multi-step process have elucidated how leukocytes interact with endothelial cells to initiate TEM [21–27], but the exact mechanisms by which adherent leukocytes ultimately transmigrate across the endothelial barrier remain incompletely understood.

One of the key regulators of TEM is the endothelial adherens junction [28–33]. Endothelial cells form dynamic junctions that regulate the passage of molecules and cells between the circulating blood and tissues. The morphology of endothelial junctions varies both *in vivo* throughout the vascular tree and *in vitro* in endothelial cells from different organs and cultured in different conditions. In cultured quiescent cells immunostained to identify VE-cadherin, the adherens junctions display a linear distribution or appear as a reticular network along the cell-cell borders [34], a morphology likely caused by cell-cell overlap. Upon stimulation by inflammatory cytokines such as TNFα and thrombin, the adherens junctions can assume a distorted “zigzag” appearance, reflecting the tension exerted on the junctions by actin bundles. These tension-modified junctions recruit vinculin and have been referred to as “focal adherens junctions” [35]. The stability of adherens junctions can be influenced by many factors such as cytoskeletal tethering to the junctions, phosphorylation status of junction proteins, and mechanotransduction signaling [28, 30, 36]. Changes in junction stability further affect the barrier function and permeability of endothelial cells. Inflammatory stimuli can influence TEM by changing endothelial barrier function. Interestingly, all TAM kinases have been suggested to participate in regulation of endothelial permeability. AXL is essential for VEGF-A-induced permeability in endothelial cells [37, 38], and activation of TYRO3 by its ligand Protein S facilitates maintenance of the blood brain barrier during ischemia [39]. MERTK has also been implicated in maintenance of the blood brain barrier during viral infection [40]. Given that TAM kinase functions often overlap [6, 41] and MERTK is expressed in the endothelial cells [5, 42, 43], we investigated the possible involvement of MERTK in endothelial permeability and TEM.

Specifically, we examined MERTK function in normal and inflammatory conditions using an *in vitro* model of cultured human endothelial cells and an *in vivo* mouse model of acute pneumonia. We tested the hypothesis that MERTK-mediated signaling regulates two aspects of the inflammatory process, neutrophil recruitment and endothelial permeability. Our *in vitro* findings demonstrate that endothelial MERTK contributes to maintaining proper endothelial barrier function by regulating neutrophil TEM and endothelial permeability. Our *in vivo* results show that global deletion of MERTK in mice leads to a more severe inflammatory response to acute *P*. *aeruginosa* pneumonia, enhancing neutrophil recruitment to the lungs and vascular permeability in the lungs. In contrast, endothelial cell-specific deletion of MERTK alone does not result in this enhanced inflammatory response.

## Materials and methods

### Endothelial cell culture

Human lung microvascular endothelial cells (ECs) isolated from two healthy donors (one 7-year-old female and one 9-year-old female) were purchased from Lonza (HMVEC-L, CC-2527, Lot-0000677243 & Lot-0000318000) and cultured according to manufacturer instructions. MV2 medium from Promo Cell (C-39221) and Lonza (CC-4147) were used. Post-transfection medium was normal MV2 medium with 10% FBS and no antibiotics. For all experiments except the XPerT assay, culture dishes or wells were coated overnight at 37°C with 10μg/mL human fibronectin (FN) resuspended in PBS. ECs were used up to passage 6.

### Antibodies and reagents

MERTK and AXL siRNA oligonucleotides were obtained from Santa Cruz (SC-37127, SC-29769) and from Sigma [44–46]. The sequences of all siRNA oligonucleotides and the control siRNA against luciferase are shown in **Table 1**. Western blot antibodies for MERTK (4319), AXL (8661), TYRO3 (5585), and ICAM-1 (4915) were purchased from Cell Signaling. Western blot antibodies for PECAM-1 (376764), VCAM-1 (8304), E-selectin (137054), and VE-cadherin (9989) were purchased from Santa Cruz. Western blot antibody for actin (1501) was purchased from Millipore and western blot antibody for Rac1 (610651) was purchased from BD. The antibodies used for flow cytometry are listed in **Table 2**. Human fibronectin (354008) was purchased from Corning. Oligofectamine (12252–011) and Calcein-AM dye (C34852) were purchased from Thermo Fisher Scientific. Opti-MEM (31985–070), DPBS (14190–144), RPMI medium 1640 (11875–093), and Trypsin-EDTA (25300–054) were purchased from Gibco. Trypsin Neutralizer Solution (CC-5002) was purchased from Lonza. TNFα (210-TA) was purchased from R&D Systems. N-Formylmethionyl-leucyl-phenylalanine (fMLP) was purchased from Sigma (F3506). 70kD FITC-conjugated dextran was purchased from Invitrogen (1823), resuspended in PBS at 12.5mg/mL, and stored at -20°C. DNase I (LS002140) and collagenase type I (LS004197) used for lung digestion were purchased from Worthington.

**Table 1:** siRNA sequences.

siRNA molecule	Sense sequence	Antisense sequence
Ctrl (Luciferase)	CGUACGCGGAAUACUUCGAdTdT	UCGAAGUAUUCCGCGUACGdTdT
Mer-A	CCAUCUACAUCGAAGUACAdTdT	UGUACUUCGAUGUAGAUGGdTdT
Mer-B	CCACAUCUGUACCAAAUCAdTdT	UGAUUUGGUACAGAUGUGGdTdT
Mer-C	CUAGGUGUGUGUAUAGAAAdTdT	UUUCUAUACACACACCUAGdTdT
Axl-A	CCACUGAAGCUACCUUGAAdTdT	UUCAAGGUAGCUUCAGUGGdTdT
Axl-B	CAGCGAGAUUUAUGACUAUdTdT	AUAGUCAUAAAUCUCGCUGdTdT
Axl-C	CUGUGAGUCUUUGGUUCUAdTdT	UAGAACCAAAGACUCACAGdTdT

The sequences of siRNA molecules targeting MERTK (Mer-A, Mer-B, and Mer-C) and AXL (Axl-A, Axl-B, Axl-C) were obtained from Santa Cruz. siRNA targeting luciferase was used as control (Ctrl) siRNA.

**Table 2:** Flow cytometry antibodies and their targeted cell populations.

Antibody	Targeted Cells	Company	Catalog #
APC anti-mouse CD31, clone 390	Endothelial Cells	BioLegend	102410
Pacific blue anti-mouse CD45, clone 30F11	Leukocytes	BioLegend	103126
APC/Cy7 anti-mouse Ly-6G, clone 1A8	Neutrophils	BioLegend	127624
Zombie Aqua Dye	Dead cells	BioLegend	423101
PE anti-mouse MERTK antibody, clone 2B10C42	All cells expressing MERTK	BioLegend	151505

### siRNA knockdown experiments

ECs were cultured on 10cm dishes to 60% confluency. For each 10cm plate, 15μL of 20μM siRNA oligos were mixed with 22.5μL Oligofectamine and 820μL Opti-MEM. The mixture was incubated at room temperature for 25 minutes as reported. ECs were washed once with Opti-MEM and left in 4mL of Opti-MEM for the addition of siRNA mix. After incubation with siRNA for 4–5 hours at 37°C, 8mL of post-transfection medium was added to each 10cm plate. ECs were cultured with siRNA at 37°C for 69–72 hours.

### Cell lysates and immunoblotting

ECs were lysed in 2X sample buffer (200mM Tris pH 6.8, 20% glycerol, 5% B-mercaptoethanol, 4% SDS, 0.03% bromophenol blue) and boiled for 10 minutes. Lysates were loaded into polyacrylamide SDS-PAGE gels for protein separation by electrophoresis. The proteins were then transferred to 0.45μm PVDF membrane (Millipore IPVH00010) at 100V room temperature in transfer buffer (192mM glycine, 25mM Tris, 20% methanol) for 60–90 minutes. Membranes were blocked in TBST (50mM Tris, 150mM NaCl, 0.1% Tween-20, pH7.6) containing either 2% BSA or 5% milk at room temperature for 2 hours before being incubated with primary antibodies overnight at 4°C. Membranes were then washed, incubated with secondary antibodies, and developed as previously described [25].

### Neutrophil isolation

Human neutrophils were isolated from peripheral blood from healthy donors as previously described [47, 48]. All studies received approval from the University of North Carolina at Chapel Hill Institutional Review Board. Briefly, blood was drawn into BD vacutainer tubes (362761) containing sodium citrate and centrifuged at 1500g room temperature for 20 minutes. The cell layer containing both neutrophils and red blood cells (RBCs) was collected. RBCs were lysed by resuspension in cold RBC lysis buffer (155mM NH_4_Cl, 10mM KHCO_3_, 0.1mM EDTA, pH 7.4) and incubation on ice for 25 minutes. Purified neutrophils were then washed 2X with cold RBC lysis buffer and 1X with cold PBS before being resuspended in a small volume of RPMI medium containing 10% FBS for further use. To fluorescently label live neutrophils, purified neutrophils were resuspended at 10^6^ cell/mL in RPMI medium containing 10% FBS. 5μL of 1mM calcein-AM in DMSO was added for each 1mL of neutrophils (10^6^ cells). These cells were incubated in the dark at room temperature for 5 minutes followed by washing 1X with 10-15mL of PBS. Labelled cells were resuspended in RPMI medium containing 10% FBS for further use.

### TEM live microscopy

After 69–72 hours of siRNA treatment, ECs were trypsinized and replated onto FN-coated 35mm glass-bottom dishes at confluent density (450k cells per dish). At 6–7 hours post replating, ECs were treated with 10ng/mL TNFα for 18–20 hours and transferred to a Viva View FL Incubator Microscope (Olympus) for phase contrast imaging. Freshly isolated neutrophils were activated by 10-minute-incubation at 37°C. Neutrophils (2.2 x 10^5^ cells/dish) were added to each 35mm dish, and neutrophil TEM was imaged every 15–30 seconds for 30 minutes.

### TEM in transwells

After 69–72 hours of siRNA treatment, ECs were trypsinized and replated onto FN-coated 8μm-pore, 6.5mm transwells (Corning 3422) at confluent density (1.5 x 10^5^ cells/transwell). At 6–7 hours post replating, ECs were treated with 10ng/mL TNFα for 18–20 hours. Freshly isolated neutrophils labeled with calcein-AM were activated by a 10-minute-incubation at 37°C. MV medium containing 10μM fMLP, a neutrophil chemoattractant, was added to the lower chamber of each transwell to induce neutrophil TEM. Neutrophils (2.0 x 10^5^ cells/transwell) were added to the upper chamber of each transwell and allowed to transmigrate for 30 minutes. The number of neutrophils that transmigrated through the EC monolayer was quantified by measuring calcein-AM fluorescence intensity in medium from the lower chamber via EnSpire 2300 Multilabel Reader (PerkinElmer). For each experiment, the medium from 5–6 transwells per condition was sampled and plotted.

### Dextran permeability

After 69–72 hours of siRNA treatment, ECs were trypsinized and replated onto FN-coated 0.4μm-pore, 6.5mm transwells (Corning 3470) at confluent density (1.5 x 10^5^ cells/transwell). At 6–7 hours post replating, ECs received either normal MV2 medium or MV2 medium containing 10ng/mL TNFα, and were cultured for another 19 hours. Afterwards, the medium in the upper chamber was replaced with medium containing 1mg/mL FITC-conjugated 70kD dextran. After 90 minutes, medium in the lower chamber was sampled, and FITC fluorescence intensity was measured at 488nm via EnSpire 2300 Multilabel Reader (PerkinElmer).

### XPerT assay and image analysis

15mm glass coverslips were coated with biotinylated gelatin as previously described [49, 50]. These coverslips were then coated with FN as described above before the experiment. After 69–72 hours of siRNA treatment, ECs were trypsinized and replated onto these coverslips at confluent density (2.5–3.0 x 10^5^ cells/coverslip). At 6–7 hours post replating, ECs received either normal MV2 medium or MV2 medium containing 10ng/mL TNFα, and were cultured in the given medium for 19–20 hours. To measure permeability, MV2 medium containing 1:500 diluted FITC-conjugated streptavidin (Thermo Fisher Scientific S11223) was added to each coverslip (1mL per coverslip) and cells were incubated at 37°C for 2 minutes before being washed once and then fixed with 3.7% formaldehyde/PBS. Hoechst dye (1:20,000) was used to stain for nuclei. Coverslips were imaged at 10X in the 488nm channel for FITC-streptavidin and the 350nm channel for Hoechst using a Zeiss epifluorescence microscope. In each experiment, two coverslips were plated per condition, and four imaging fields were randomly picked from each coverslip for image analysis. Images were then assigned a random file name for blinded analysis. The percentage of FITC-streptavidin positive area and the number of nuclei per imaging field were quantified with ImageJ.

### Immunofluorescence

After 69–72 hours of siRNA treatment, ECs were trypsinized and replated onto FN-coated 15mm glass coverslips at confluent density (2.5–3.0 x 10^5^ cells/coverslip). At 6–7 hours post replating, ECs received either normal MV2 medium or MV2 medium containing 10ng/mL TNFα, and then were cultured for 19–20 hours. ECs were washed once and then fixed with 3.7% formaldehyde/PBS for immunofluorescence staining. After permeabilizing with 2% Triton-X/TBS and blocking with 2% BSA/TBS, ECs were first stained with VE-cadherin antibody (Santa Cruz 9989, 1:500), followed by anti-mouse secondary antibody (1:250), phalloidin (1:500), and Hoechst dye (1:20,000). Coverslips were imaged at 20X in the 568nm channel for actin, 488nm channel for VE-cadherin, and the 350nm channel for Hoechst using a Zeiss epifluorescence microscope. In each experiment, two coverslips were plated per condition.

### Rac1 assay

After 69–72 hours of siRNA treatment, ECs were trypsinized and replated onto FN-coated 6cm dishes at confluent density (1.5–1.6 x 10^6^ cells/dish). At 26–27 hours post replating, ECs were lysed to assay Rac1 activity as previously described [25].

### Gene array analysis

Human ECs were grown on fibronectin-coated plates for 76 hours before mRNA was collected. Microarray analysis was carried out using the Affymetrix HuGene 2.1 array. Array quality was evaluated using ThermoFisher/Affymetrix Transcriptome Analysis Console v4.0 software (Grand Island, NY) based on summary statistics. All samples passed all quality control metrics. Expression signals from CEL files were preprocessed and normalized using Affymetrix Power Tools (APT), by Robust Multiarray Average (RMA) background correction, GC content correction, quantile normalization, and median polish summarization of probe signals mapped to specific genes. Custom probeset-to-gene mappings in the form of meta-probeset (.mps) files were generated based on ENSEMBL release 91 transcript remaps of Affymetrix probesets.

### Tamoxifen oil preparation

Tamoxifen (Sigma, T-5648) was resuspended in sterile corn oil (Sigma, C-8267) at a concentration of 20mg/mL and was rotated at 37°C overnight. The resuspended tamoxifen oil was kept at 4°C and used for up to a month.

### Mice

Adult C57BL/6 (WT) mice were originally purchased from Jackson Laboratory (Bar Harbor, ME), and the colony was bred in the UNC facility. Mice deficit in *Mertk* were generated by introducing a neomycin resistance cassette into exon 18, the last exon encoding the 3' end of the kinase domain [10, 11]. These *Mertk*^*-/-*^ mice are catalogued in Jackson Laboratory (Stock No. 011122) and were obtained from the Earp lab colony. Floxed *Mertk* (*Mertk*^*fl/fl*^) mice were generated for the Earp lab by the UNC Lineberger Animal Models Core Facility. Mice expressing the tamoxifen-inducible Cre-recombinase (Cre-ERT2) under the regulation of the vascular endothelial cadherin promoter (VECad), denoted Cdh5-CreERT2, were originally generated by Monvoisin et al. [51] and are available at Taconic (Model #13073). These mice were kindly provided by Dr. James E. Faber (Department of Cell Biology and Physiology, University of North Carolina Chapel Hill). The primer sequences for genotyping these mice are provided in **Table 3**. Mice were bred and housed side by side in microisolator cages within ventilated racks in a specific pathogen-free facility.

**Table 3:** The sequences of PCR primers for each of the three transgenic mouse lines.

Primer	Sequence	PCR Product Size		
**floxed Mer**		**WT**	**loxP / WT**	**loxP / loxP**
5’ loxP_Forward	ATTAGAGATTCAAAGATGGA		217 bp	217bp
5’ loxP_Reverse	TTTACTATAGTATGAATTCACTGA	177bp	177bp	
3’ loxP_Forward	CAATGCCCATTCCCT		228 bp	228 bp
3’ loxP_Reverse	CCAGTCTGGAATGGGTC	188bp	188bp	
**Full Mer knockout**		**WT**	**heterozygotes**	**Full Mer KO**
Mut Forward	ATC AGC AGC CTC TGT TCC AC		250bp	250bp
WT Forward	TGC CAT TAT ACC TGG CTT TCA	235bp	235bp	
Common Reverse	CAT CTG GGT TCC AAA GGC TA			
**Inducible VE-cadherin Cre**		**Cre-/Cre-**	**Cre+/Cre-**	**Cre+/Cre+**
Cre Forward	GACCAGGTTCGTTCACTCA			
Cre Reverse	TAGCGCCGTAAATCAAT	no band	~400bp	~400bp
RAP Forward	AGGACTGGGTGGCTTCCAACTCCCAGACAC			
RAP Reverse	AGCTTCTCATTGCTGCGCGCCAGGTTCAGG	~600bp	~600bp	~600bp

A tamoxifen-inducible endothelial-specific *Mertk*^*-/-*^ *(*iEC *Mertk*^*-/-*^*)* mouse line was generated by crossing the Cdh5-CreERT2 mice with the *Mertk*^*fl/fl*^ mice. Cre- littermates of the Cre+ iEC *Mertk*^*-/-*^ mice were used as controls. To induce expression of Cre, mice of both genotypes (Cre- and Cre+) received 2mg tamoxifen per day (intraperitoneal injection of 100μL of 20mg/mL stock) for 5 consecutive days, beginning at age 4–6 weeks. Mice were closely monitored daily and provided with easier access to food and water during the injection period. Experiments with the Cre+ iEC *Mertk*^*-/-*^ male mice and Cre- littermates were carried out 11–16 days (6 to 8 weeks of age) after the first tamoxifen injection. Age-matched male wild type (WT) and *Mertk*^*-/-*^ mice were used at 6 to 8 weeks of age for studies of pneumonia.

All animal studies were performed in compliance with the U.S. Department of Health and Human Services Guide for the Care and Use of Laboratory Animals. Animal studies were reviewed and approved by our Institutional Animal Care and Use Committee. For the acute pneumonia experiments carried out in this study, animals were monitored at 15min, 1h, and 2h following instillation of *P*. *aeruginosa*. Health and well-being was assessed at each time point by evidence of distress, as documented by high respiratory rates, ruffled hair, lethargy, and decreased activity.

### Initiation of bacterial pneumonia

Gram-negative bacteria *Pseudomonas aeruginosa* (strain PA01) were grown at 37°C with 5% CO_2_ on trypticase soy agar containing 5% sheep blood (BD 221261) for 16–18 hours prior to experiments. Suspensions of bacteria were prepared in sterile PBS at a concentration of 0.1 OD absorbance measured at 600nm via Genesys 10UV Spectrophotometer (Thermo Fisher Scientific). Pneumonia was induced by intratracheal instillation of the prepared bacterial suspension into the left lung (2.27 μL of the bacterial suspension per gram of mouse body weight). Male mice of age 6 to 8 weeks and weight 18 to 25g were studied. The number of bacterial colony-forming units (CFU) instilled was quantified by plating serial dilutions of the bacterial suspension in sterile PBS on trypticase soy agar containing 5% sheep blood. Bacterial colonies were counted on culture plates after overnight incubation at 37°C and 5% CO_2_. The range of CFU instilled was 1.02–1.62 x 10^8^ CFU/mL instillate or 5.2–8.7 x 10^6^ CFU/mouse.

### Blood sampling and bronchoalveolar lavage

Mice were euthanized by isoflurane overdose, and blood (0.2–0.5mL) from the inferior vena cava was obtained using heparin-coated 25G needles (BD 305122) and syringes (BD 309659). Bronchoalveolar lavage (BAL) was performed as described [52, 53]. Briefly, 0.9 mL of ice-cold PBS containing 2mM EDTA was instilled into the lungs via a 24G intratracheal catheter (BD 381412), and the fluid was carefully aspirated. The lavage was repeated another five times with fresh buffer and the fluid samples were pooled. Leukocyte concentration in the blood and the BAL fluid (BALF) were determined using a hemocytometer. Differential counts were determined using blood smears and cytospins [54] stained with Hema3 (Thermo Fisher Scientific 22–122911) by a researcher blinded to the sample genotypes.

### Total protein concentration in BALF

Total protein concentration in the 1st BALF was analyzed using the Bicinchoninic Acid Protein Assay (BCA) as previously described [52].

### *In vivo* permeability assay in lungs

A modified Miles assay was used to assess vascular permeability in the lungs [55–58]. Evans blue dye (200μL per mouse, 0.5% in PBS) was injected into the tail vein of 6–8 week old male mice weighing between 18-25g. At the time specified for each experiment, mice were euthanized by isoflurane overdose and blood (0.3–0.5mL) from inferior vena cava was sampled. The pulmonary vasculature of each mouse was perfused with cold PBS containing heparin (7.5mL/min) through the right ventricle until the lungs were white/gray. Then the lungs were excised, and Evans blue in the lungs was extracted using 1mL formamide overnight at 75°C. Blood samples were centrifuged at 3000 rpm for 10 minutes at 4°C to obtain plasma. Evans blue absorbance in PBS-diluted plasma (1:200) was measured at 620nm (E620) and 740nm (E740) using a spectrophotometer (Genesys 10UV Spectrophotometer, Thermo Fisher Scientific). After formamide extraction, lung samples were centrifuged at 13,000g for 30 minutes. Evans blue absorbance in the supernatant was measured at 620nm and 740nm. The following formula was used to correct optical densities (E) for contamination with heme pigments: E620 (corrected) = E620 - (1.426 x E740 + 0.030). Evans blue permeability in the lungs was calculated as the ratio of E620 (corrected) from the lungs to E620 (corrected) from the plasma.

### Preparation of single-cell suspension of lung tissues for flow cytometry

Male mice of age 6 to 8 weeks and weight 18 to 25g were studied. Mice were euthanized by isoflurane overdose, and the lung vasculature was flushed as described above. Cold lung digestion buffer (RPMI containing 0.1% DNase and 0.5% collagenase) was instilled into the trachea (1mL/mouse), and the lungs were excised as previously described [52–54]. Enzymatic digestion was performed 37°C for 30 minutes, followed by mincing with forceps and mechanical disruption using an 18G needle and syringe. The cells were filtered through 100μm filters and then processed as previously described [52, 53].

### Immunostaining and flow cytometry

Isolated single lung cell suspensions were incubated with antibodies listed in **Table 3** at 4°C for 30 minutes as previously described [52]. After incubation with the appropriate antibodies, cells were washed once with staining buffer (PBS containing 1.6% BSA and 2mM EDTA) before loading on the CytoFLEX Flow Cytometer (Beckman Coulter) for flow cytometry analysis. Data obtained from these experiments were analyzed with CytExpert 2.1 software (Beckman Coulter).

### Statistics

Groups were compared using a two-tail Student T-test or one-way ANOVA with Tukey post hoc adjustment in R, as appropriate. Data from *in vitro* experiments are presented as mean ± SEM bar graphs unless otherwise specified. Data from *in vivo* experiments are presented as dot plots overlaid with box plot, where the mean is the horizontal line inside the box, the ends of the box are mean ± SD, and the whiskers extended to the highest and the lowest of the values in the group. Differences between groups were considered significant when p<0.05.

## Results

### TAM kinases are expressed in ECs

**Fig 1:**
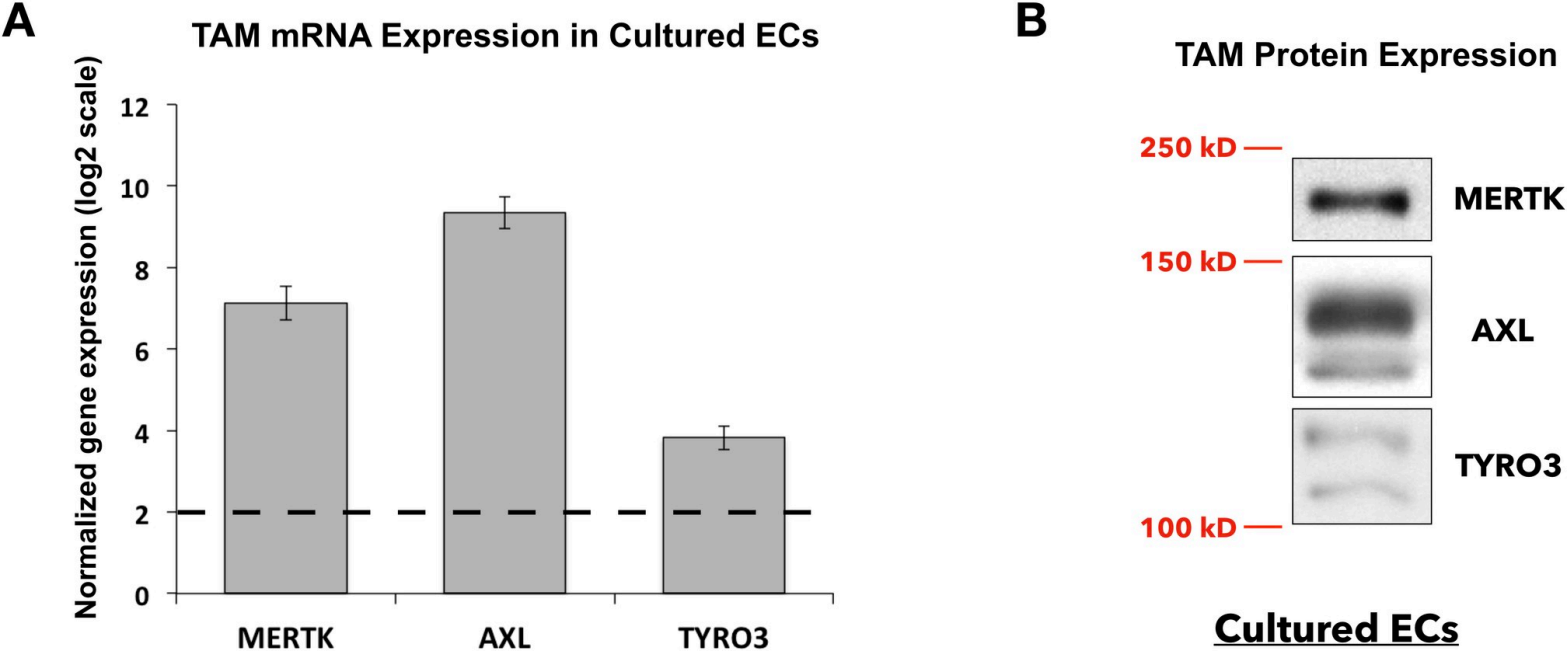
Expression of the three TAM kinases in cultured human lung microvascular endothelial cells (ECs). **A**, TAM family kinase (TYRO3, AXL, MERTK) mRNA expression levels in cultured ECs from gene array analysis. Dashed line (log_2_) indicates the background threshold. **B**, MERTK, AXL, and TYRO3 protein expression in lysates from cultured confluent ECs. Lysate loading was increased 3–4 fold for the TYRO3 blot, compared to the loading amount used for the MERTK and AXL blots. Relevant molecular weight annotations (250 kD, 150kD, 100kD) are shown in red.

Gene array analyses of TAM kinase expression in ECs detected all three TAM kinases at the mRNA level (Fig 1A). Expression of TAM kinases in ECs was further confirmed by examining TAM protein levels in total cell lysates (Fig 1B). Because of difficulty in detecting TYRO3 from cell lysates, we pursued studies of MERTK and AXL in pulmonary microvascular endothelial cells during inflammation.

### MERTK depletion increases neutrophil TEM *in vitro*

**Fig 2:**
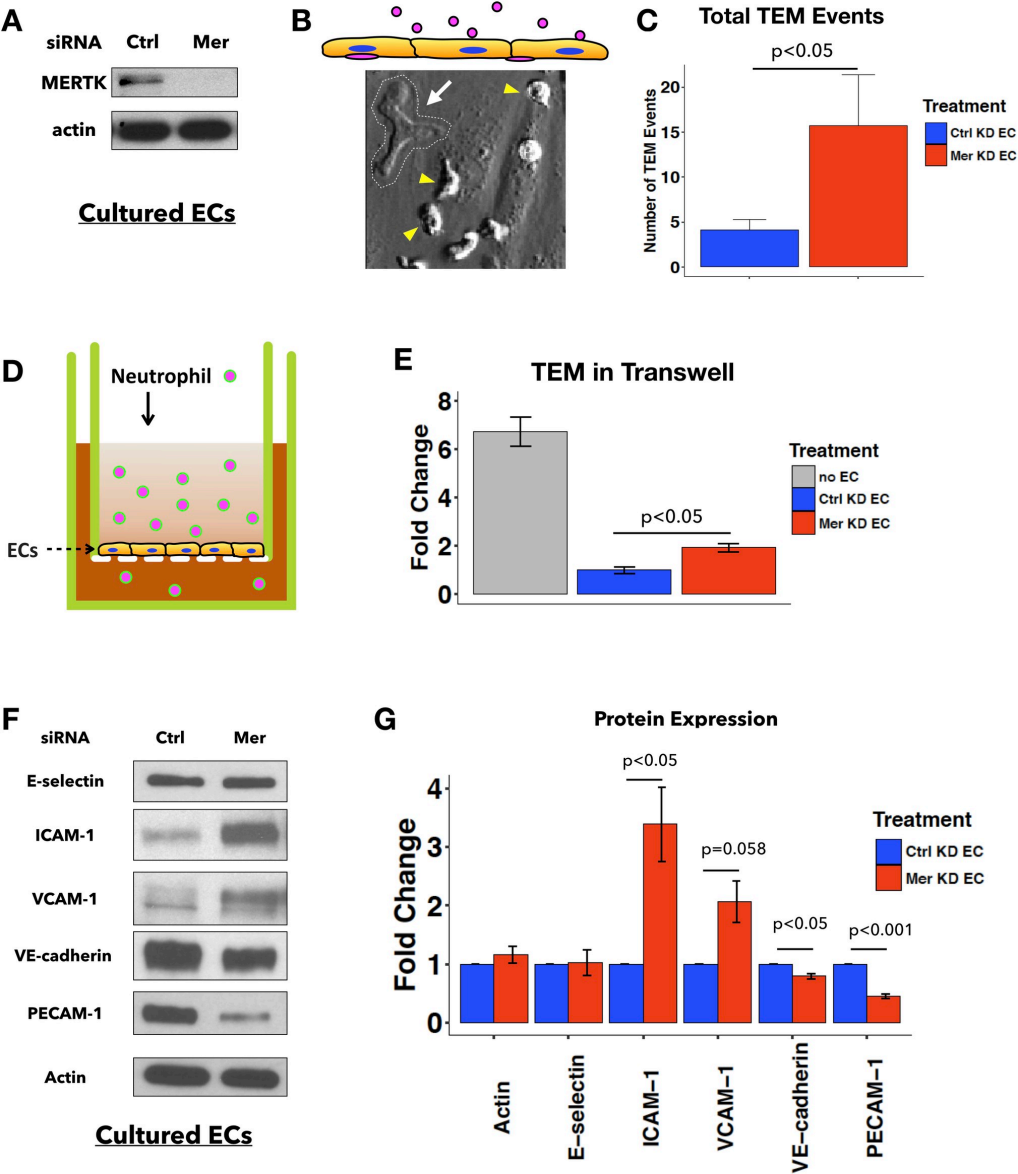
EC MERTK inhibits neutrophil TEM *in vitro*. **A**, Efficient reduction of MERTK expression in ECs was achieved by siRNA. **B**, Schematic diagram of the neutrophil TEM assay and a sample image during time-lapse microscopy. In the schematic diagram (top), ECs are labeled in yellow and neutrophils are labeled in pink (cells are not drawn to scale). In the sample image during TEM (bottom), a confluent monolayer of ECs pretreated with TNFα overnight makes up the image background. A transmigrated neutrophil (white arrow), outlined by a white dashed line, appears flattened and phase-dark compared to the untransmigrated neutrophils that remain on the apical side of the EC monolayer (yellow arrowheads). **C**, Quantification of the total number of TEM events per imaging field (n = 12 for Ctrl KD and n = 11 for Mer KD). Data are pooled from 3 independent experiments. 2-sample student T test was used for statistical analysis. **D**, Schematic diagram of Transwell TEM assay. Confluent monolayers of ECs were cultured in the upper chamber on a porous membrane support and stimulated with TNFα overnight. Fluorescently labeled neutrophils were added to the upper chamber, and fMLP (10μM), a neutrophil chemokine, was added to the bottom chamber. Medium from the bottom chamber was sampled after 30 minutes, and fluorescence was measured as an indicator of neutrophil TEM. **E**, Quantification of neutrophil TEM expressed as fold change. Fluorescence intensity for all conditions was normalized to Ctrl KD ECs. The positive control was the absence of EC monolayers on the filter (no EC). Total number of transwells: n = 11 for no EC, n = 24 for Ctrl KD EC and for Mer KD EC. Data are combined from 4 independent experiments. One-way ANOVA with post hoc Tukey test (p < 0.05) was used for statistical analysis. **F**, Representative immunoblots of E-selectin, ICAM-1, VCAM-1, VE-cadherin, PECAM-1, and actin in whole cell lysates. ECs pretreated with indicated siRNA oligos were replated at confluent density and cultured in normal medium overnight before being lysed for immunoblotting. **G**, Densitometric quantification of protein expression level of actin, E-selectin, ICAM-1, VCAM-1, PECAM-1 and VE-cadherin. Graph represents fold change normalized to values from Ctrl KD EC in each experiment (n = 4 independent experiments). 2-sample student T test was used for statistical analyses.

To examine the role of MERTK in neutrophil TEM, MERTK expression in ECs was reduced by siRNA-mediated knockdown using a pool of three different siRNA oligonucleotides (Table 1, Fig 2A and S1 Fig). After knockdown, ECs were then re-plated onto FN-coated glass bottom dishes for imaging and treated with TNFα overnight. Primary human neutrophils isolated from healthy donors were added to the EC monolayers, and TEM events were recorded by time-lapse microscopy for 30 minutes (Fig 2B and S1 Movie). Interestingly, MERTK depletion in ECs (Mer KD EC) led to increased neutrophil TEM compared to ECs treated with control siRNA (Ctrl KD EC) (Fig 2C). Increased neutrophil TEM in MERTK-depleted ECs was also observed in a transwell assay (Fig 2D). In this assay, a commonly used neutrophil chemoattractant, fMLP, was added to the lower chamber to stimulate neutrophil TEM across the EC monolayers. Fluorescently-labeled neutrophils were added to the apical side of TNFα-treated ECs and incubated for 30 minutes. Neutrophil TEM was quantified by accumulated fluorescence intensity of the medium containing transmigrated neutrophils in the lower chamber. Compared to the control, MERTK-depleted ECs allowed more neutrophil TEM in the transwell assay, as demonstrated by increased fluorescence intensity (Fig 2E). Taken together, these results suggest that the expression of MERTK in ECs lessens neutrophil TEM *in vitro*.

The contribution of MERTK to the expression of leukocyte adhesion molecules and junctional proteins was determined using immunoblots of control and MERTK-depleted ECs. MERTK depletion increased the expression of two key leukocyte-endothelial cell adhesion molecules, ICAM-1 and VCAM-1, but did not affect E-selectin, another important leukocyte adhesion molecule in ECs (Fig 2F and 2G). More high molecular weight VCAM-1 was detected in Mer KD cells (Fig 2F), suggesting MERTK may regulate post-translational modification of VCAM-1. In contrast, expression of VE-cadherin and PECAM-1 were reduced after MERTK depletion. These proteins are important in regulating junctional integrity. Our data suggest that important mechanisms through which MERTK may be acting include modulating the expression of adhesion molecules to decrease leukocyte adhesion and preventing neutrophil transendothelial migration by enhancing junctional integrity. Subsequent studies addressed the contributions of MERTK to junctional barrier function.

### MERTK maintains endothelial barrier function *in vitro*

**Fig 3:**
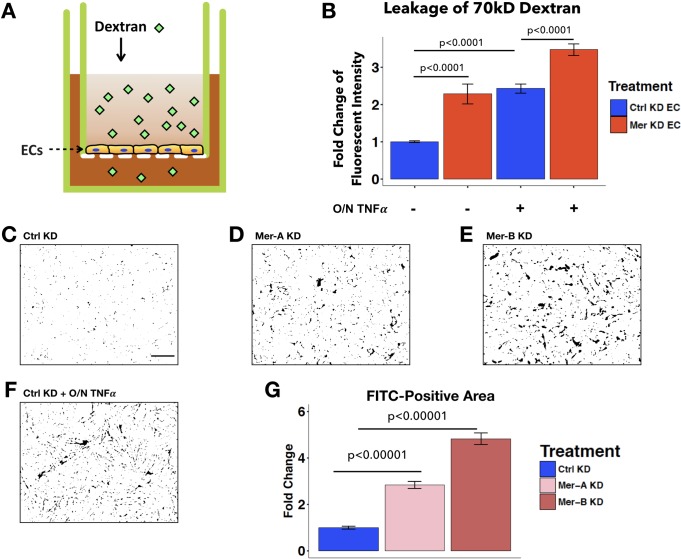
Endothelial MERTK is required for EC barrier function. **A**, Schematic diagram of transwell permeability assays. 70kD FITC-dextran was added to the upper chamber for 1.5h. Then medium from the bottom chamber was sampled and measured for fluorescence to quantify permeability. **B**, Quantification of dextran leakage expressed as fold change compared to ECs treated with control siRNA. ECs were pretreated with siRNA prior to replating on transwells at confluent density. ECs plated on transwells were either untreated or treated with TNFα overnight (O/N TNFα) as indicated, before dextran addition. Fluorescent intensity was normalized to Ctrl KD ECs without O/N TNFα treatment. Data represent 29–39 transwells per condition, combined from six independent experiments. **C-F**, Permeability test by xPerT assay. Representative thresholded images of local permeability in Ctrl KD (C), Mer-A KD (D), and Mer-B KD (E) EC monolayers, as identified by FITC-streptavidin (black) binding to exposed underlying cell substrate. Ctrl KD with O/N TNFα treatment (F) was used as a positive control for high permeability. Scale bar: 200μm. **G**, Quantification of percent FITC-positive area per imaging field, expressed as fold change normalized to Ctrl KD ECs. (n = 24 imaging fields). Results are combined from 6 coverslips per condition in 2 independent experiments. One-way ANOVA with post hoc Tukey test (p < 0.05) was used for statistical analyses.

The increased neutrophil TEM in cultured ECs depleted of MERTK led us to investigate whether EC permeability was also increased in ECs lacking MERTK. To examine endothelial permeability, we measured the passage of 70kD FITC-dextran across confluent EC monolayers grown on transwell inserts (Fig 3A) [59]. Compared to the Ctrl KD ECs, more dextran molecules passed across the Mer KD EC monolayers (Fig 3B). This increased endothelial permeability due to MERTK depletion was also observed after overnight TNFα stimulation (Fig 3B), suggesting that MERTK is important for maintaining proper barrier function during both homeostasis and inflammation.

To visually examine endothelial barrier integrity, we applied the “express micromolecule permeability testing” (XPerT) assay [49, 50]. This assay takes advantage of the high-affinity interactions between FITC-conjugated streptavidin added in the medium with exposed biotinylated gelatin substrate at sites of localized permeability. MERTK depletion in ECs via two different single siRNA oligos (Mer-A KD and Mer-B KD) resulted in a higher percentage of the coverslips being streptavidin-positive compared to control (Ctrl KD) (Fig 3C–3G), suggesting that permeability is increased by MERTK depletion. Equal cell seeding density in all conditions was confirmed (S2 Fig). KD by single siRNAs (Mer-A or Mer-B) achieved similar level of MERTK depletion as the pooled Mer siRNA used for previous experiments (S1 Fig) and published by other studies [44].

**Fig 4:**
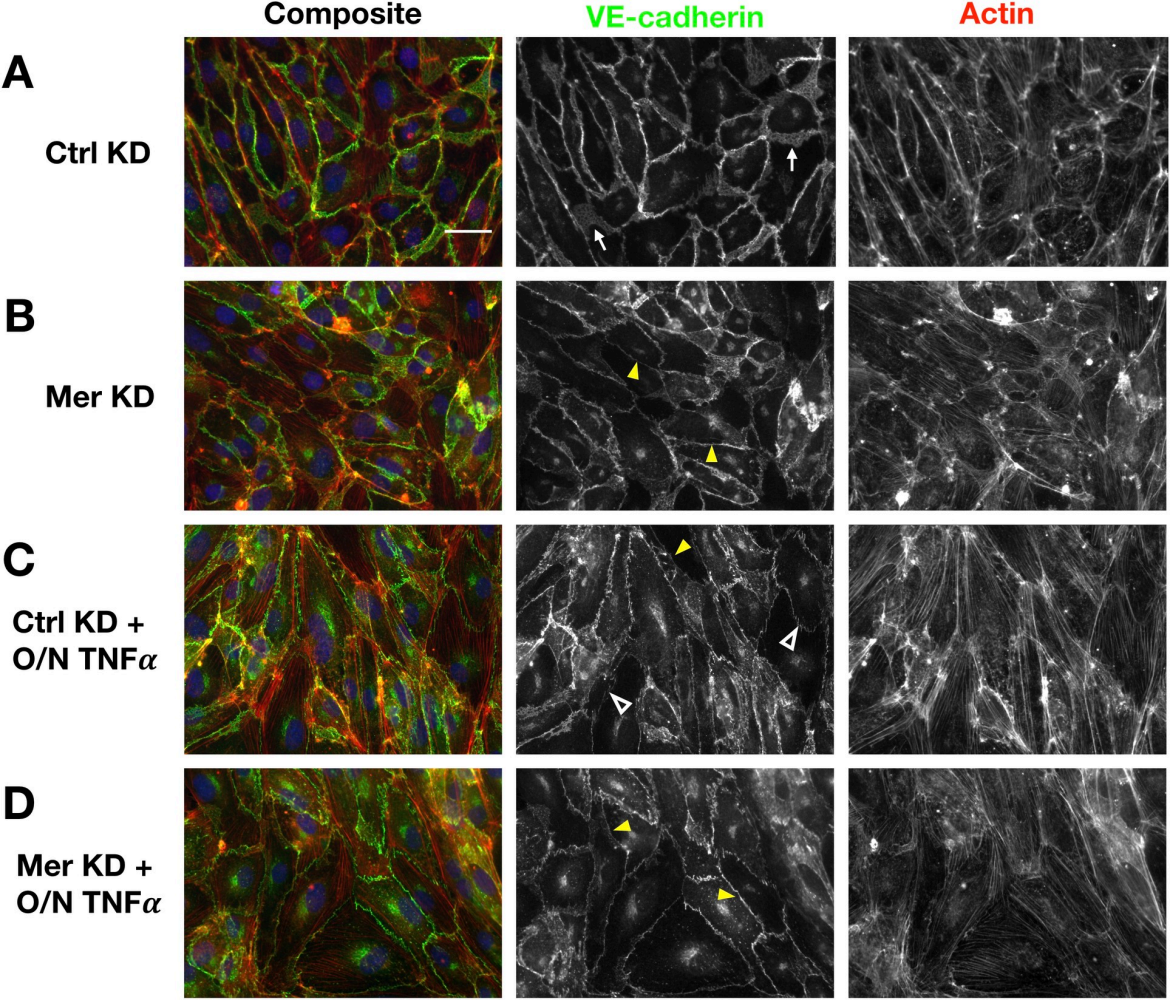
Endothelial MERTK depletion reduced reticular junction formation heterogeneously *in vitro*. **A-D**, Representative immunofluorescence images of VE-cadherin (green) and actin (red) in Ctrl KD ECs (A), Mer KD ECs (B), Ctrl KD with O/N TNFα treatment (C), and Mer KD with O/N TNFα treatment (D). White arrows point to VE-cadherin in reticular junctions (A), yellow arrowheads point to VE-cadherin in linear junctions (B-D), and empty white arrowheads point to VE-cadherin in focal adherens junctions (C). Scale bar: 40μm.

Taking a closer examination at the endothelial junctions, we stained VE-cadherin and actin in ECs. Compared to Ctrl KD ECs, Mer KD ECs displayed far more heterogeneity in junctional VE-cadherin levels. Some Mer KD ECs had notably decreased VE-cadherin at the junctions, yet no obvious lack of cell-cell contact was detected in unstimulated Mer KD ECs using immunofluorescence microscopy (Fig 4A and 4B). Whereas Ctrl KD ECs exhibited reticular junction morphology as defined by Fernández-Martín et. al. in many cells across the monolayer [34], fewer Mer KD ECs had the reticular junction morphology (Fig 4B). In regions of Mer KD ECs where VE-cadherin intensity was decreased, the junctions were mostly linear (Fig 4B). In TNFα-treated cells, ECs depleted of MERTK lacked the typical TNFα-induced elongated cell shape and discontinuous junction morphology that Huveneers et. al. described as “focal adherens junctions” (Fig 4C and 4D) [35]. Taken together, these results suggest that MERTK plays an important role in regulating endothelial junction structures and permeability, both at resting conditions and after TNFα stimulation.

### Examining the role of AXL in ECs

**Fig 5:**
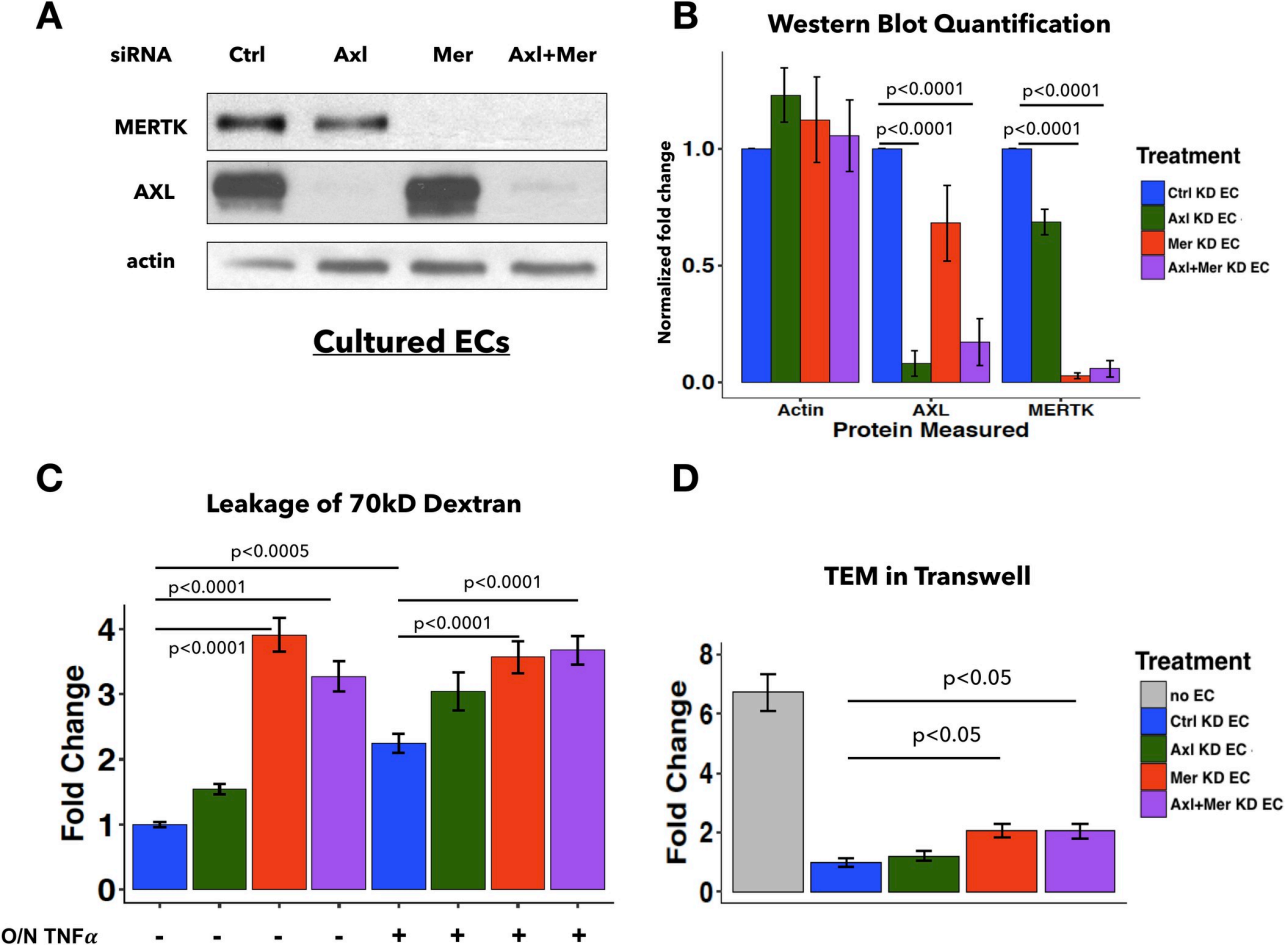
AXL depletion in ECs *in vitro* does not affect endothelial permeability or neutrophil TEM. **A**, Expression of MERTK and/or AXL in ECs was efficiently and specifically reduced by siRNA KD. Actin was used as a loading control. **B**, Densitometric quantification of AXL and MERTK protein level from Ctrl KD EC, Axl KD EC, Mer KD EC, and Axl+Mer KD EC conditions. Graph represents fold change normalized to Ctrl KD EC condition (n = 6 independent experiments). One-way ANOVA with post hoc Tukey test was used for statistical analyses. **C**, Quantification of dextran leakage, expressed as fold change compared to Ctrl KD ECs (n = 17–18 transwells per condition). Data are combined from three independent experiments. ECs were treated with indicated siRNA prior to replating onto transwells at confluent density. ECs plated on transwells were either untreated or treated with TNFα overnight (O/N TNFα) as indicated, before dextran addition. Fluorescent intensity was normalized to Ctrl KD ECs. **D**, Quantification of neutrophil TEM expressed as fold change (total number of transwells: n = 11 for no EC, n = 18 for all other groups). Data are combined from three independent experiments. Fluorescent intensity was normalized to Ctrl KD ECs. The positive control was the absence of EC monolayers on the filter (no EC). One-way ANOVA with post hoc Tukey test was used for statistical analyses.

TAM kinases often have overlapping functions in various types of cells [5–7, 41]. Other studies have demonstrated that AXL contributes to increased endothelial permeability caused by VEGF signaling [37, 38], which contrasts with the role of MERTK as a suppressor of EC barrier function seen here in our studies. The third member of the TAM kinase family, TYRO3, was difficult to detect in ECs. Consequently, we only investigated the role of AXL in TEM and endothelial barrier function. Knockdown experiments against AXL (Axl, a pool of three different siRNA oligos), MERTK (Mer), a combination of AXL and MERTK (Axl+Mer), and control siRNAs in ECs were performed. Efficiency of AXL and/or MERTK knockdown was confirmed by immunoblot analysis (Fig 5A and 5B). FITC-dextran permeability assays revealed that, unlike Mer KD, Axl KD did not significantly increase dextran permeability compared to Ctrl KD in ECs either with or without overnight TNFα stimulation (Fig 5C). XPerT assay also revealed that Axl KD did not significantly increase FITC-conjugated streptavidin-positive area across EC monolayers (S3 Fig). Additionally, Axl KD did not increase neutrophil TEM in the transwell assay, as did Mer KD (Fig 5D). Combined Axl+Mer KD led to phenotypes more similar to those caused by Mer KD than by Axl KD, and had no additive effect compared to Mer KD alone (Fig 5C and 5D). These results suggest that in cultured ECs, MERTK plays a major role in regulating EC barrier function under unstimulated and inflammatory conditions, whereas AXL does not.

### MERTK depletion in ECs reduces basal Rac1 activity

**Fig 6:**
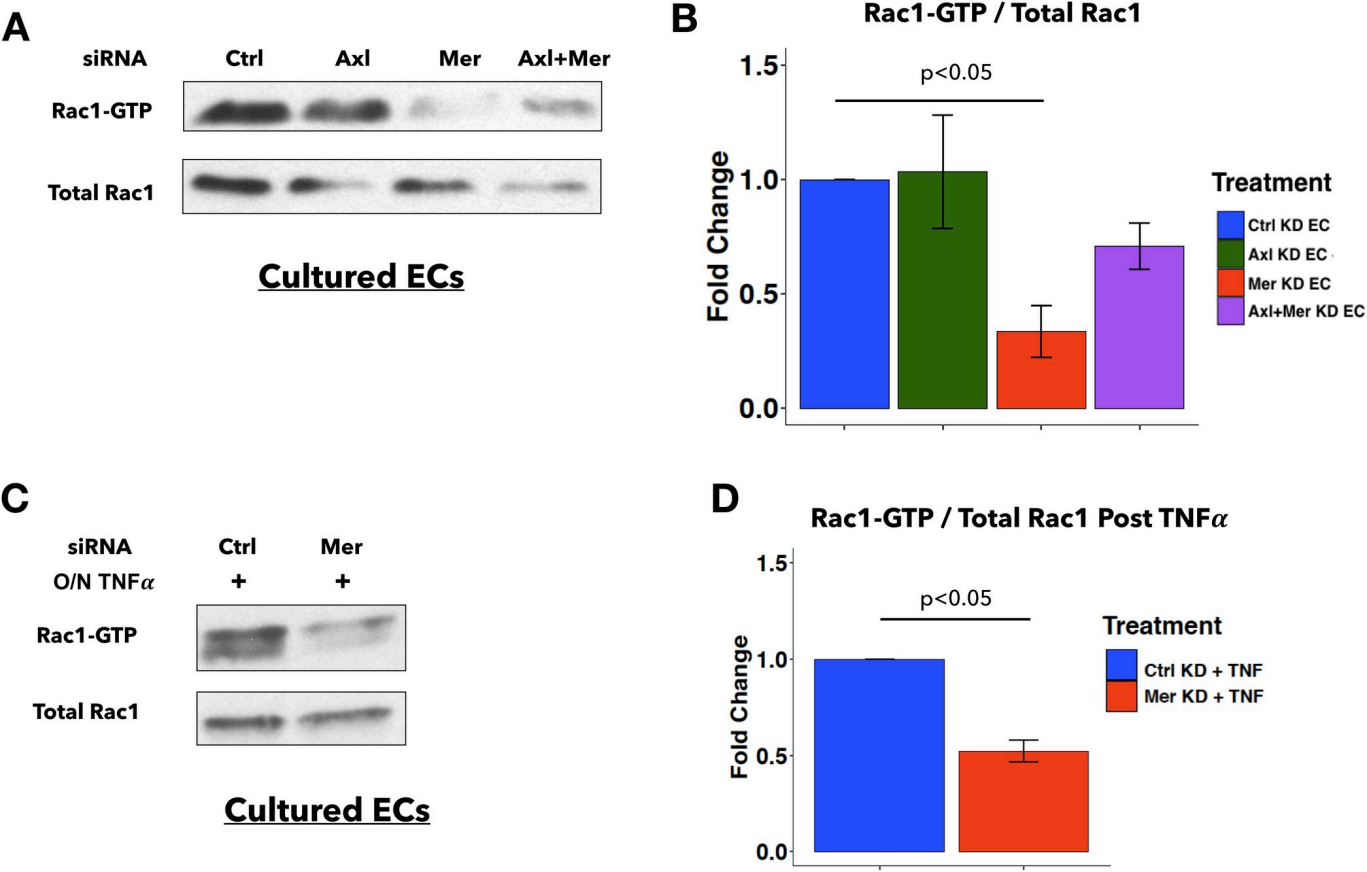
MERTK depletion in ECs *in vitro* reduces basal Rac1 activity. **A**, Representative immunoblots from assays measuring active Rac1 (Rac1-GTP), compared to total Rac1 in whole cell lysates. ECs pretreated with indicated siRNA oligos were replated at confluent density for 27h before performing Rac1 activity assays. **B**, Densitometric quantification of Rac1-GTP (active Rac1) to total Rac1 ratio (active/total). Values represent fold change normalized to Ctrl KD EC in each experiment (n = 3 independent experiments). One-way ANOVA with post hoc Tukey test was used for statistical analyses. **C**, Representative immunoblots from assays measuring active Rac1 (Rac1-GTP) compared to total Rac1 in whole cell lysates. ECs pretreated with indicated siRNA oligos were replated at confluent density and cultured in TNFα-containing medium overnight before performing Rac1 activity assays. **D**, Densitometric quantification of Rac1-GTP (active Rac1) to total Rac1 ratio (active/total) after overnight TNFα treatment. Values represent fold change normalized to Ctrl KD EC post overnight TNFα treatment in each experiment (n = 3 independent experiments). 2-sample student T test was used for statistical analyses.

To understand the mechanisms through which MERTK may be acting in ECs, we examined signaling downstream of MERTK in ECs. Previous work showed that MERTK regulates the activity of the small GTPase Rac1 in monocytes [19]. Since Rac1 is one of the key regulators of EC junctions and leukocyte TEM [25, 30, 60], we examined the effect of MERTK depletion on Rac1 activity in ECs. When MERTK expression was reduced via siRNA knockdown, Rac1 basal activity was decreased (Fig 6A and 6B). By contrast, AXL depletion did not significantly decrease Rac1 activity (Fig 6A and 6B). The effect of MERTK depletion on Rac1 basal activity persisted after TNFα stimulation, since less active Rac1 was detected in Mer KD ECs compared to Ctrl KD ECs after overnight TNFα treatment (Fig 6C and 6D). Given that high Rac1 activity is associated with tighter EC junctions and enhanced barrier function [60–62], our finding that MERTK regulates Rac1 activity in ECs provides one mechanism mediating the increased permeability in MERTK-depleted ECs.

### TNFα affects MERTK and AXL expression in ECs

Inflammatory cytokines can regulate TAM kinase expression in macrophages [63]. To determine whether the inflammatory stimulus used in our study, TNFα, affects MERTK and AXL expression, we compared their protein levels before and after overnight TNFα treatment. While Mer KD consistently reduced MERTK expression in ECs treated with or without TNFα, TNFα stimulation alone decreased MERTK and increased AXL expression (S4 Fig).

### Global MERTK depletion in mice leads to greater inflammatory response during pneumonia

**Fig 7:**
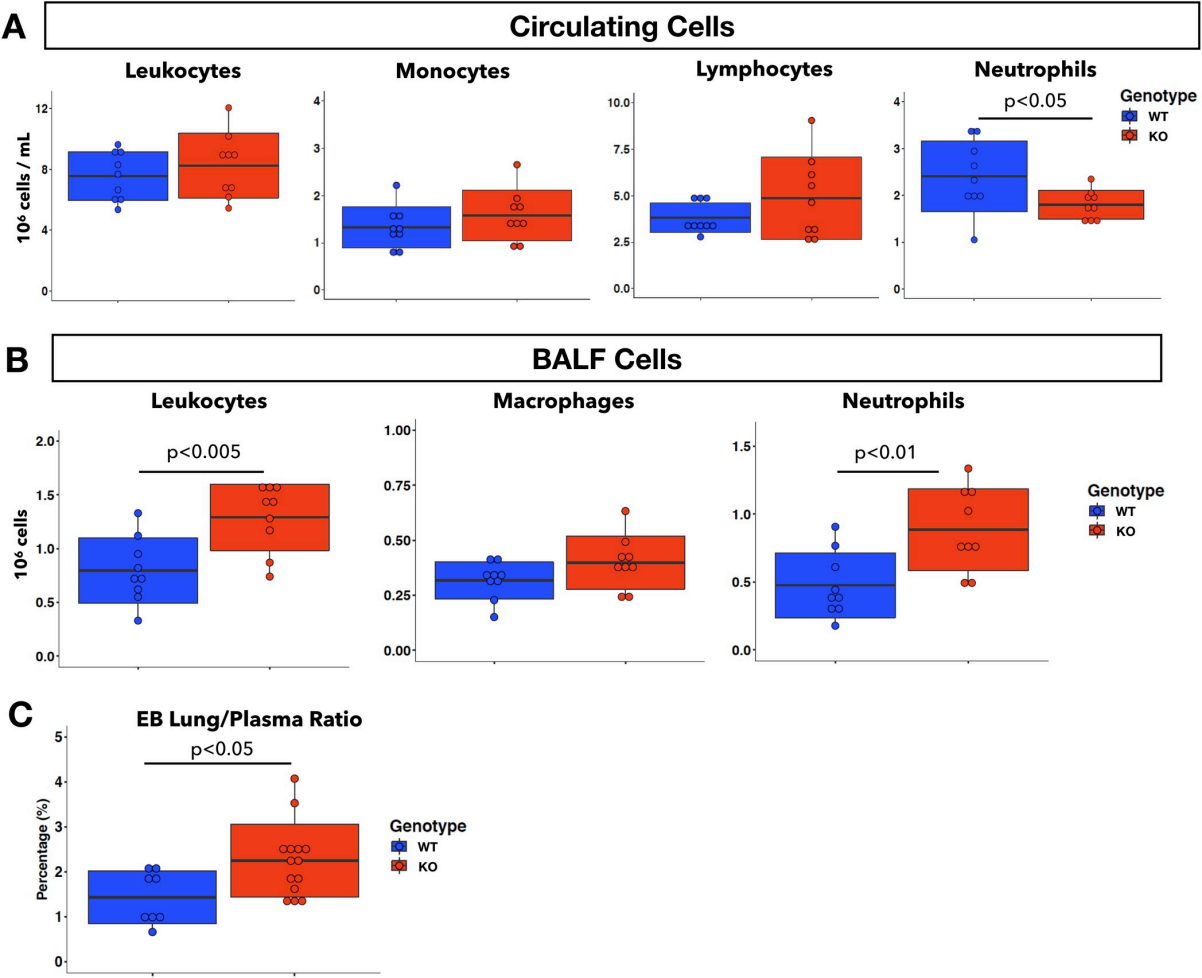
Global *Mertk*^*-/-*^ mice have increased neutrophil infiltration and endothelial permeability during 4h *P*. *aeruginosa* pneumonia. **A**, Concentration (10^6^ cells /mL) of circulating leukocytes and subtypes (monocytes, lymphocytes, and neutrophils) from WT and global *Mertk*^*-/-*^ (KO) mice at 4h after initiation of pneumonia. **B**, Total cell count (10^6^ cells) of leukocytes and subtypes (macrophages, and neutrophils) in BALF obtained from WT and *Mertk*^*-/-*^ (KO) mice at 4 hpi. n = 9 per group. **C**, Quantification of Evans blue (EB) leakage into the lungs expressed as the ratio of EB absorbance measured in whole lung tissues over EB absorbance measured in the plasma from WT and *Mertk*^*-/-*^ (KO) mice between 3h to 4h pneumonia (n = 8 for WT, n = 14 for KO; data were pooled from two independent experiments). Two-tail student t test was used for statistical analyses.

Our *in vitro* results demonstrated that MERTK is an important regulator of endothelial barrier function as it acts to block neutrophil TEM and EC permeability. To examine the role of MERTK *in vivo*, we challenged global *Mertk*^*-/-*^ mice in our *P*. *aeruginosa* infection model. At 4 hours post-infection (hpi) with *P*. *aeruginosa*, *Mertk*^*-/-*^ mice had similar levels of circulating leukocytes, monocytes, and lymphocytes compared to WT (Fig 7A). However, circulating neutrophils were decreased (Fig 7A). In the lungs, there were more leukocytes in the BALF collected from *Mertk*^*-/-*^ compared to WT mice, and this increase was due to an increase in neutrophils (Fig 7B). BALF macrophages were similar between the two genotypes (Fig 7B). To measure pulmonary vascular permeability during pneumonia, we intravenously injected Evans blue dye at 3 hpi and harvested the lungs at 4 hpi (S5A Fig). *Mertk*^*-/-*^ mice demonstrated increased Evans blue leakage into the lungs between these time points than WT mice (Fig 7C). Unchallenged *Mertk*^*-/-*^ mice showed no change in pulmonary permeability compared to WT mice (S5B Fig), consistent with previous findings that single TAM knockout mice are viable and superficially healthy [10, 11]. These results suggest that global deletion of MERTK, which is known to produce a hyperinflammatory state [10], results in increased infiltration of neutrophils into the lungs, leading to fewer neutrophils in the circulation, and enhanced vascular permeability.

### Endothelial-specific MERTK depletion in mice does not alter the acute inflammatory response

**Fig 8:**
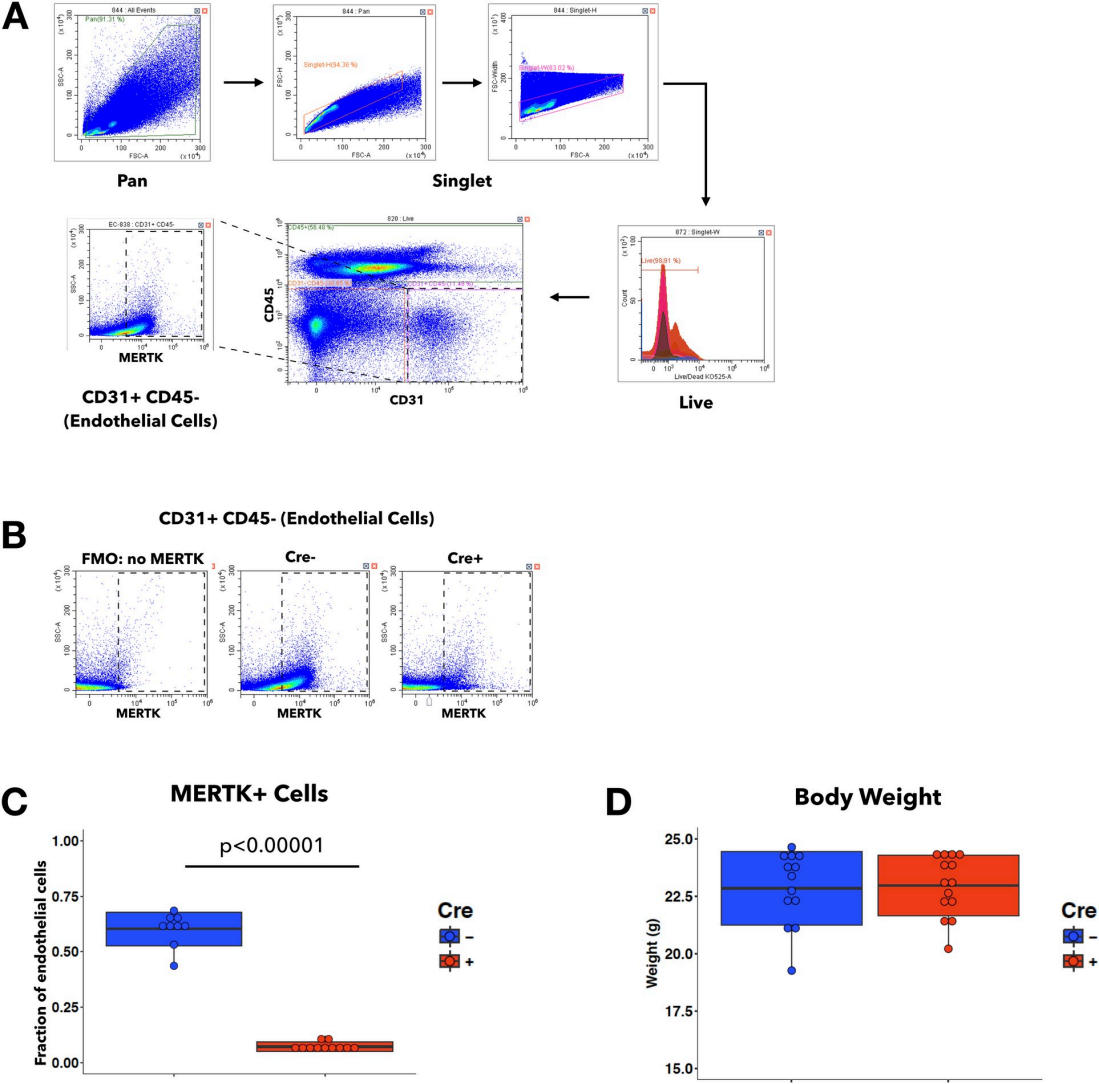
Endothelial MERTK deletion in iEC *Mertk*^*-/-*^ mice. **A**, Representative images and gating strategies of flow cytometry analyses to isolate EC population (CD31+CD45-) from the whole lung digest. After singlet cells were identified, dead cells were excluded. By gating on CD31 and CD45, we identified the CD31+CD45- population as the EC population. The expression of surface MERKT was then assessed on ECs. **B**, Representative images of MERTK staining in CD31+CD45- EC population. Panels (left to right): fluorescence minus one control (FMO: no MERTK), Cre-, and Cre+ mice. **C**, Quantification of MERTK-positive EC fraction from Cre- and Cre+ mice (n = 9 Cre-; n = 11 Cre+). **D**, Body weight of Cre- and Cre+ mice aged between 6 to 8 weeks (n = 13 Cre-; n = 14 Cre+).

To examine whether the aggravated inflammatory response to pneumonia observed in *Mertk*^*-/-*^ mice was caused by specific depletion of MERTK in endothelial cells, we generated a tamoxifen-inducible endothelial-cell specific *Mertk*^*-/-*^ mouse line *(*iEC *Mertk*^*-/-*^*)*. Cre- littermates of the Cre+ iEC *Mertk*^*-/-*^ mice served as controls. After tamoxifen injection, MERTK expression in endothelial cells was measured by flow cytometry (Fig 8A–8C). MERTK expression was decreased by 87% in the endothelial cells of Cre+ mice compared to their Cre- littermates (Fig 8C). Both Cre- and Cre+ mice had similar weights (Fig 8D) at 7 weeks of age and appeared healthy. There was no difference in Evans blue dye leakage into lung tissues of unchallenged Cre- and Cre+ mice (S5C Fig), suggesting that in uninjured mice, there was no difference in pulmonary vascular permeability, similar to the case of global *Mertk*^*-/-*^ mice.

**Fig 9:**
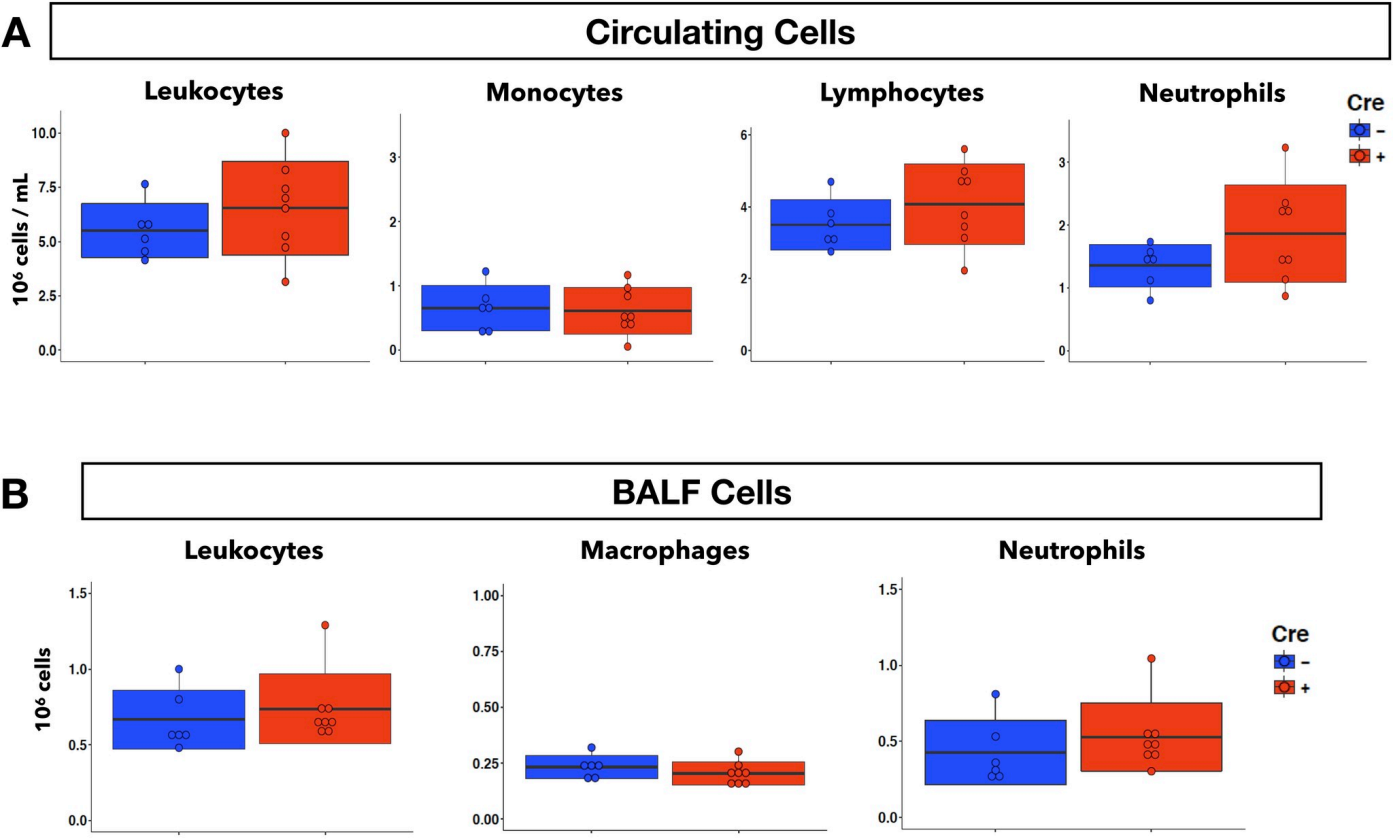
Endothelial-specific MERTK deletion does not increase neutrophil infiltration into the lungs during 4h *P*. *aeruginosa* pneumonia in mice. **A**, Concentration (10^6^ cells /mL) of circulating leukocytes and subtypes (monocytes, lymphocytes, and neutrophils) from Cre- and Cre+ iEC *Mertk*^*-/-*^ mice at 4h after initiation of pneumonia. **B**, Total cell count (10^6^ cells) of leukocytes and subtypes (macrophages, and neutrophils) in BALF obtained from Cre- and Cre+ iEC *Mertk*^*-/-*^ mice at 4h pneumonia. (n = 6 Cre-, n = 8 Cre+ mice; data were pooled from two experiments). Two-tail student T test was used for statistical analyses.

**Fig 10:**
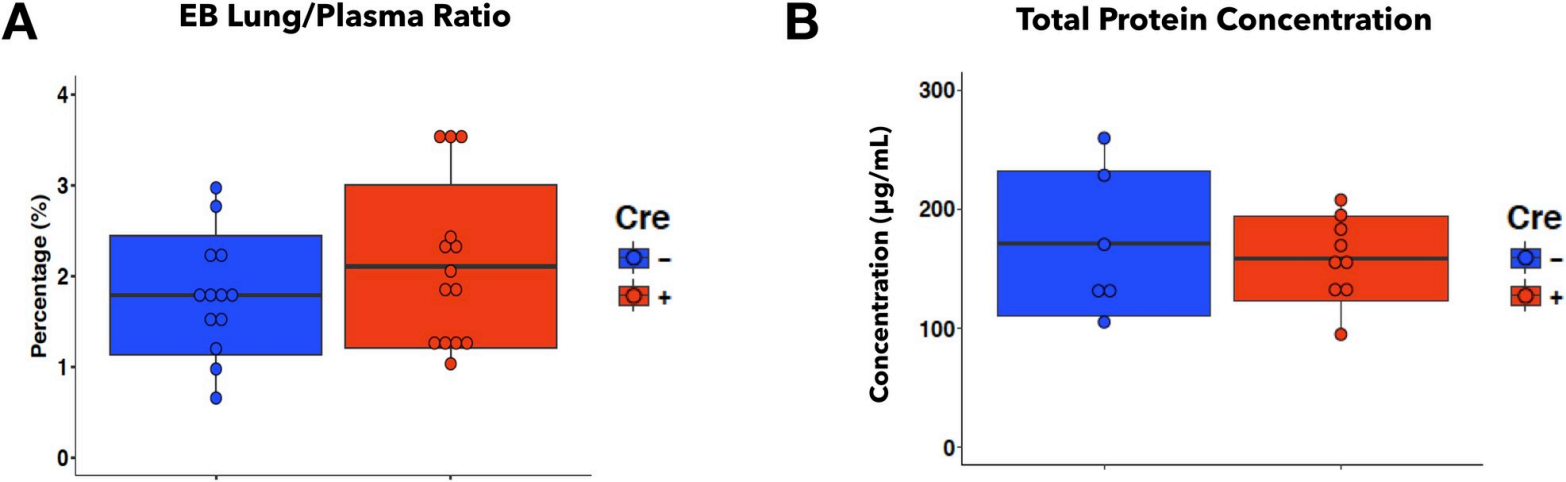
Endothelial-specific MERTK deletion does not increase endothelial permeability in the lungs during 4h *P*. *aeruginosa* pneumonia in mice. **A**, Quantification of Evans blue (EB) leakage into the lungs expressed as the ratio of EB absorbance measured in whole lung tissues over EB absorbance measured in the plasma from Cre- and Cre+ iEC *Mertk*^*-/-*^ mice between 3h to 4h pneumonia (n = 13 Cre-, n = 14 Cre+ mice; data were pooled from three independent experiments). **B**, Quantification of total protein concentration in BALF obtained from Cre- and Cre+ iEC *Mertk*^*-/-*^ mice at 4h pneumonia (n = 6 Cre-; n = 9 Cre+ mice; data were pooled from two experiments). Two-tail student T test was used for statistical analyses.

To determine if Cre- and Cre+ iEC *Mertk*^*-/-*^ mice would respond differently to inflammatory stimulus, we challenged them in our acute *P*. *aeruginosa* pneumonia model. At 4 hpi, the number of circulating leukocytes and subtypes in response to acute pneumonia was similar between the two genotypes (Fig 9A). Analysis of BALF showed no difference in total leukocyte, neutrophil, or macrophage recruitment to the airways and alveolar space of the lungs in Cre- and Cre+ iEC *Mertk*^*-/-*^ mice (Fig 9B). This was further confirmed by whole lung digestion and immuno-phenotyping via flow cytometry, which quantifies all neutrophils in the lung tissue (S6 Fig). Evans Blue permeability assay revealed that both Cre- and Cre+ iEC *Mertk*^*-/-*^ mice had similar pulmonary vascular permeability in response to *P*. *aeruginosa* pneumonia (Fig 10A). Additionally, total protein concentration in the BALF obtained from mice at 4 hpi was also similar in Cre- and Cre+ iEC *Mertk*^*-/-*^ mice (Fig 10B). Taken together, these results suggest that while global depletion of MERTK worsens the pulmonary inflammatory response to *P*. *aeruginosa*, deleting MERTK in endothelial cells alone does not alter this response in murine lungs.

## Discussion

Herein, we report that *in vitro*, MERTK contributes to endothelial barrier function in unstimulated ECs and after TNFα, modulating both neutrophil TEM and endothelial permeability. When MERTK was depleted from ECs, more neutrophil TEM occurred and endothelial permeability was increased. MERTK depletion also increased the expression of two adhesion molecules, ICAM-1 and VCAM-1, and decreased the expression of two junctional proteins, VE-cadherin and PECAM-1, in ECs. The decreased VE-cadherin level upon MERTK knockdown corresponded to a heterogeneous junction morphology that is distinct from control: some Mer KD ECs presented more linear and fewer reticular junctions compared to Ctrl KD ECs. These observations suggest that MERTK may regulate endothelial barrier function by modulating junction formation and stability in ECs. In contrast, depletion of AXL, another TAM kinase that exhibits overlapping functions with MERTK in various cell types [5, 13], did not alter either neutrophil TEM or endothelial permeability. Ruan and colleagues showed previously that AXL is required for the increase in endothelial permeability that occurs downstream of VEGF signaling, and its absence prevents this increase [37, 38]. These data suggest that MERTK and AXL regulate endothelial permeability differently. In addition to the opposing roles of attenuating versus enhancing permeability, there seem to be other differences in the types of responses they modulate. For example, AXL may be more important during angiogenesis, whereas MERTK may regulate permeability during homeostasis and inflammation. The contribution of TYRO3, the third member of the TAM family, to maintaining proper blood brain barrier under hypoxic conditions [39] further suggests that TAM kinases take on unique roles in regulating EC functions. However, we did not investigate the role of TYRO3 because we had difficulty detecting it in ECs.

One potential mechanism through which MERTK regulates endothelial barrier function is by modulating junction architecture. Endothelial adherens junctions mediate adhesion between neighboring cells via homophilic interactions of VE-cadherin and associated cortical actin bundles [30]. One type of adherens junctions, the reticular adherens junctions, are maintained by PECAM-1 and contribute to endothelial barrier function [34]. Both PECAM-1 and VE-cadherin were reduced in Mer KD ECs (Fig 2F and 2G). Additionally, we observed that some regions in the MERTK-depleted EC monolayers displayed fewer reticular junctions (Fig 4B). These observations suggest that MERTK is involved in retaining barrier-enhancing reticular junctions via maintaining PECAM-1 and VE-cadherin levels in ECs. This barrier-protective function is important in limiting endothelial permeability *in vitro*.

In addition to modulating junction protein expression, MERTK may also maintain endothelial barrier function by regulating Rac1 activity. Rac1-GTP level was reduced in ECs lacking MERTK but not AXL. These findings are consistent with a previous study in monocytes, which reported that MERTK regulates Rho family GTPases via interacting with the GEF Vav1 to reorganize the cytoskeleton [19]. Since physiological activation of Rac1 is important in maintaining the peripheral actin cytoskeletal network and junction integrity [61, 64], decreased Rac1 activity upon MERTK depletion may lead to enhanced endothelial permeability. Timmerman et. al. also pointed out a link between Rac1 activity and junctional VE-cadherin stabilization [61]. The N-terminal GEF domain of Trio that activates Rac1 is required to prevent nascent VE-cadherin junctions from disassembling in endothelial cells. Additionally, Trio is highly associated with junctional VE-cadherin in recently confluent endothelial cells [61], a condition similar to that used in our experiments. This study poses interesting questions about whether the reduced VE-cadherin level, reticular junctions, and Rac1 activity in MERTK-depleted endothelial cells are interconnected events.

The role of MERTK in maintaining the endothelial barrier in homeostasis and in attenuating neutrophil TEM and endothelial permeability during TNF-induced inflammation is interesting in light of other studies investigating the function of endothelial MERTK. Fraineau and colleagues show that, in human umbilical vein endothelial cells, endothelial MERTK mediates protein S-induced activation of SHP2 and inhibition of VEGF-A–stimulated proliferation [42]. They document a protein S/Mer/SHP2 axis that inhibits VEGFR2 signaling, suggesting protein S as an endogenous angiogenesis inhibitor via binding to MERTK [42]. Using *Mertk*^*-/-*^ mice, Miner and colleagues show that MERTK maintains the blood-brain barrier during viral infection with either West Nile or La Crosse encephalitis viruses [40]. They provide evidence that the mechanism is through enhancing Type I interferon signaling [40]. A series of papers by Zhen, Shao, and colleagues show that MERTK is expressed on glomerular endothelial cells and it downregulates renal inflammation induced by nephrotoxic serum [65–67]. They identified several mechanisms through which MERTK tames inflammation, such as suppressing Stat1 and Stat3 expression, reducing ERK1/2 and Akt activation, inhibiting NF-*k*B, and promoting SOCS3 expression [66]. These scattered reports in different organs and inflammatory processes come together with our studies to suggest that endothelial MERTK regulates inflammatory processes and endothelial repair.

In global *Mertk*^*-/-*^ mice, the lack of MERTK resulted in increased neutrophil infiltration and pulmonary vascular permeability during acute *P*. *aeruginosa* pneumonia. These important inflammatory parameters indicate that *Mertk*^*-/-*^ mice develop a more severe inflammatory response to *P*. *aeruginosa* than WT mice. Thus, our *in vivo* data are consistent with our *in vitro* results demonstrating a role for MERTK in suppressing neutrophil TEM and changes in permeability.

In endothelial-specific *Mertk*^*-/-*^ (iEC *Mertk*^*-/-*^) mice, however, we did not observe increased neutrophil infiltration or pulmonary vascular permeability in response to *P*. *aeruginosa* compared to Cre- controls. Neither neutrophil migration into the alveolar space nor into the pulmonary parenchyma was changed by deficiency of endothelial MERTK. These findings are in contrast to our *in vitro* results, where MERTK strikingly suppresses TEM and permeability in ECs. This discrepancy may result from several differences between our *in vivo* and *in vitro* studies. First, differences between human and murine endothelial cells must always be considered, though studies from two different groups have suggested a similar suppressive role for MERTK during inflammation in human endothelial cells and in primary endothelial cells isolated from *Mertk*^*-/-*^ mice [40, 66]. Second, differences in the junctional complexes between *in vitro* cultures and *in vivo* pulmonary endothelium are certain to be present. This may contribute particularly to the observed role for MERTK in homeostasis only within the cultured ECs. Additionally, the complexity of *in vivo* anatomy and physiology in a live organ that contains multiple cell types and performs complicated functions contrasts with studying MERTK in one cell type cultured on two-dimensional dishes. *In vivo*, the endothelial cells are architecturally supported by other cell types, including fibroblasts and epithelial cells that communicate with the endothelial cells. Third, the *in vitro* assays used neutrophils isolated and perhaps altered during their purification from human blood samples, whereas *in vivo*, the leukocytes had not been manipulated at all. Purification may result in increased phosphatidylserine on the neutrophil surface, which can interact with MERTK. Fourth, other considerations are the differences in the duration of MERTK depletion, which was 72 hours in cultured human ECs and 2 weeks in the murine endothelial cells (from the start of tamoxifen injection to the initiation of pneumonia). While our *in vitro* experiments using human ECs ruled out AXL as an accessory regulator of TEM or endothelial permeability, it is possible that other signaling pathways and/or paracrine contributions from other local cell types compensated for deficiency of endothelial MERTK in murine lungs. Genetic and epigenetic compensation induced by deleterious mutations, but not siRNA-mediated knockdown should also be considered [68, 69].

Finally, in the iEC *Mertk*^*-/-*^ mice, not all endothelial cells are completely deficient in MERTK. On average in our studies, about 8% of endothelial cells in Cre+ iEC *Mertk*^*-/-*^ mice showed detectable expression of MERTK, compared to about 60% of endothelial cells in Cre- iEC *Mertk*^*-/-*^ mice (Fig 8B and 8C). It is possible that this level of expression may, in fact, be sufficient to prevent excessive neutrophil infiltration and edema. This expression may result from incomplete deletion by tamoxifen, perhaps due to the number of capillary segments that are not perfused while tamoxifen is circulating [70]. Alternatively, the observed expression of MERTK in 8% of endothelial cells from the Cre+ iEC *Mertk*^*-/-*^ mice may be due to microparticles from circulating monocytes or those that originate from alveolar macrophages but have entered the circulation [71]. These microparticles may bind to endothelial cells and result in apparent MERTK expression that is capable of mediating normal MERTK functions [72].

In summary, our studies show that expression of MERTK occurs on both human and murine pulmonary endothelial cells. *In vitro*, MERTK acts to maintain low levels of endothelial permeability, and to attenuate neutrophil TEM as well as endothelial permeability induced by TNFα. In mice, global deficiency of MERTK has no effect on the unchallenged lung endothelium, but results in enhanced neutrophil migration into the lungs and in greater pulmonary vascular permeability. These changes are not observed when MERTK is deficient in lung endothelial cells.

## Supporting information

S1 Fig siRNA knockdown of MERTK in cultured ECs. **A**, Densitometric quantification of MERTK protein level from Ctrl KD ECs and Mer KD ECs. Graph represents fold change normalized to Ctrl KD EC condition (n = 5 independent experiments). Two-tail student T test was used for statistical analysis. **B**, Efficient reduction of MERTK expression by single siRNA oligos (Mer-A KD or Mer-B KD). Actin was used as a loading control. **C**, Densitometric quantification of MERTK protein level from Ctrl KD, Mer-A KD, and Mer-B KD ECs. Graph represents fold change normalized to Ctrl KD EC condition (n = 7 independent experiments). One-way ANOVA with post hoc Tukey test was used for statistical analysis.
(TIF)

S2 Fig Equal seeding cell density confirmation for XPerT assay. **A-D**, Representative image fields from XPerT assay, showing cell nuclei (Hoechst stain) from Ctrl KD (A), two different Mer siRNA oligos: Mer-A KD (B) and Mer-B KD (C) ECs. Ctrl KD with O/N TNFα treatment (D) was used as a positive control for the XPerT assay. Scale bar: 200μm. **E**, Quantification of the number of nuclei per imaging field normalized to Ctrl KD ECs, expressed as fold change. n = 24 imaging fields pooled from 12 coverslips per condition in 2 independent experiments. One-way ANOVA with post hoc Tukey test was used for statistical analyses.
(TIF)

S3 Fig Endothelial AXL depletion in ECs did not affect endothelial permeability *in vitro*. **A-C,** Permeability test by xPerT assay. Representative thresholded images of local permeability in Ctrl KD (A), and Axl KD (B) EC monolayers, as identified by FITC-streptavidin (black) binding to exposed underlying cell substrate. Ctrl KD with O/N TNF𝜶 treatment (C) was used as a positive control for high permeability. **D-F,** Representative image fields from XPerT assay, showing EC nuclei (Hoechst stain) from Ctrl KD (D), and Axl KD (E) ECs. Ctrl KD with O/N TNFα treatment (F) was used as a positive control for the XPerT assay. Scale bars in A and D: 200μm. **G,** Quantification of percent FITC-positive area per imaging field, expressed as fold change normalized to Ctrl KD ECs. **H**, Quantification of the number of nuclei per imaging field normalized to Ctrl KD ECs, expressed as fold change. n = 12 imaging fields. Results are combined from 4 coverslips per condition in 2 independent experiments. 2-sample student T test was used for statistical analyses.
(TIF)

S4 Fig Overnight TNFα treatment affected MERTK and AXL expression in ECs *in vitro*. **A**, Representative immunoblots of MERTK, AXL, and actin in whole cell lysates. ECs pretreated with indicated siRNA oligos were replated at confluent density and cultured in normal medium or TNFα-containing medium overnight before being lysed for immunoblotting. **B**, Densitometric quantification of protein expression level of actin, MERTK, and AXL. Graph represents fold change normalized to values from Ctrl KD EC in each experiment (n = 8 independent experiments). One-way ANOVA with post hoc Tukey test was used for statistical analyses.
(TIF)

S5 Fig No endothelial permeability change is observed in the lungs of unchallenged total *Mertk*^*-/-*^ or iEC *Mertk*^*-/-*^ mice. **A**, Schematic diagram of the Evans blue assay. **B**, Quantification of Evans blue (EB) leakage into the lungs as expressed by the ratio of EB absorbance measured in whole lung tissues over EB absorbance measured in the plasma from unchallenged WT and KO mice at 3h after EB injection (n = 8 for WT, n = 10 for KO; data pooled from two independent experiments). **C**, Quantification of EB leakage into the lungs as expressed by the ratio of EB absorbance measured in whole lung tissues over EB absorbance measured in the plasma from unchallenged Cre- and Cre+ mice (n = 10 Cre-; n = 11 Cre+; data pooled from two independent experiments). Two-tail student T test was used for statistical analyses.
(TIF)

S6 Fig Flow cytometry analysis of whole lungs shows no significant difference in leukocyte or neutrophil infiltration within the lung tissue at 4 h after initiation of pneumonia in iEC *Mertk*^*-/-*^ mice. **A**, Representative images and gating strategies of flow cytometry analyses to isolate leukocyte population (CD45+) from whole lung digest. After singlet cells were identified, dead cells were excluded. By gating on CD45, we identified the CD45+ population as the leukocyte population. The expression of surface Ly6G was then assessed on leukocytes. **B**, Representative images of Ly6G staining in the CD45+ population. Panels (top to bottom) show cells from fluorescence minus one control (FMO: no Ly6G), Cre-, and Cre+ mice. **C-D**, Total cell counts of infiltrated leukocytes as identified by CD45+ staining (C), and neutrophils as identified by CD45+ Ly6G+ staining (D) from whole lung digest in Cre- and Cre+ mice. **E**, Fraction of leukocytes (to live cells) and **F**, neutrophils (to leukocytes) from whole lung digest in Cre- and Cre+ mice. n = 5 Cre-; n = 6 Cre+ mice from one experiment. Two-tail student T test was used for statistical analyses.
(TIF)

S1 Raw Images Original images of the immunoblots used in this manuscript. (PDF)

S1 Movie Representative movie of *in vitro* neutrophil TEM. (AVI)
