# Supplementary figures and images for: The role of endothelial MERTK during the inflammatory response in lungs
Source: PLoS One. 2019 Dec 5;14(12):e0225051. doi: 10.1371/journal.pone.0225051 (PMC6894824; doi:10.1371/journal.pone.0225051)

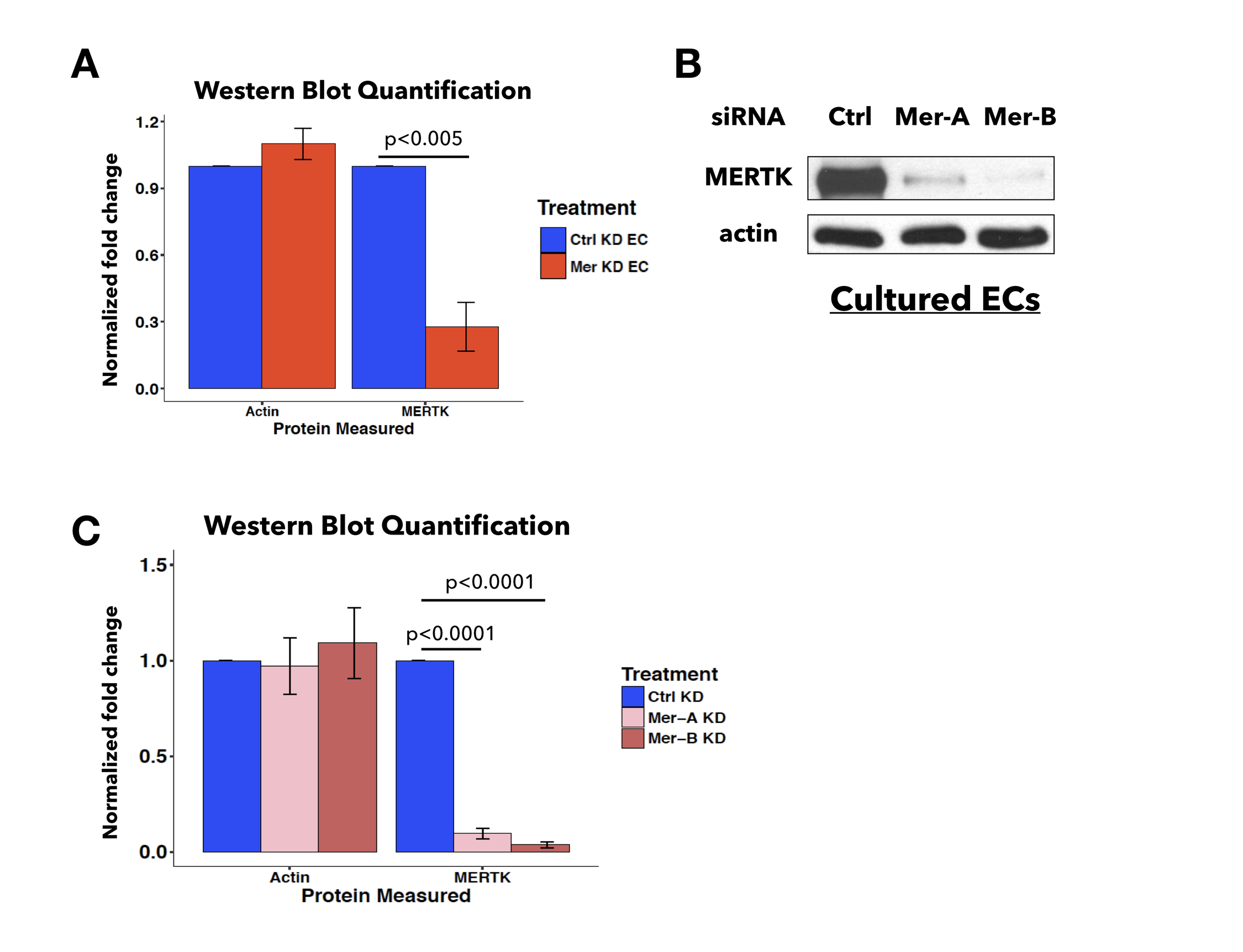

Supplement: S1 Fig — A, Densitometric quantification of MERTK protein level from Ctrl KD ECs and Mer KD ECs. Graph represents fold change normalized to Ctrl KD EC condition (n = 5 independent experiments). Two-tail student T test was used for statistical analysis. B, Efficient reduction of MERTK expression by single siRNA oligos (Mer-A KD or Mer-B KD). Actin was used as a loading control. C, Densitometric quantification of MERTK protein level from Ctrl KD, Mer-A KD, and Mer-B KD ECs. Graph represents fold change normalized to Ctrl KD EC condition (n = 7 independent experiments). One-way ANOVA with post hoc Tukey test was used for statistical analysis. (TIF) [file pone.0225051.s001.tif]

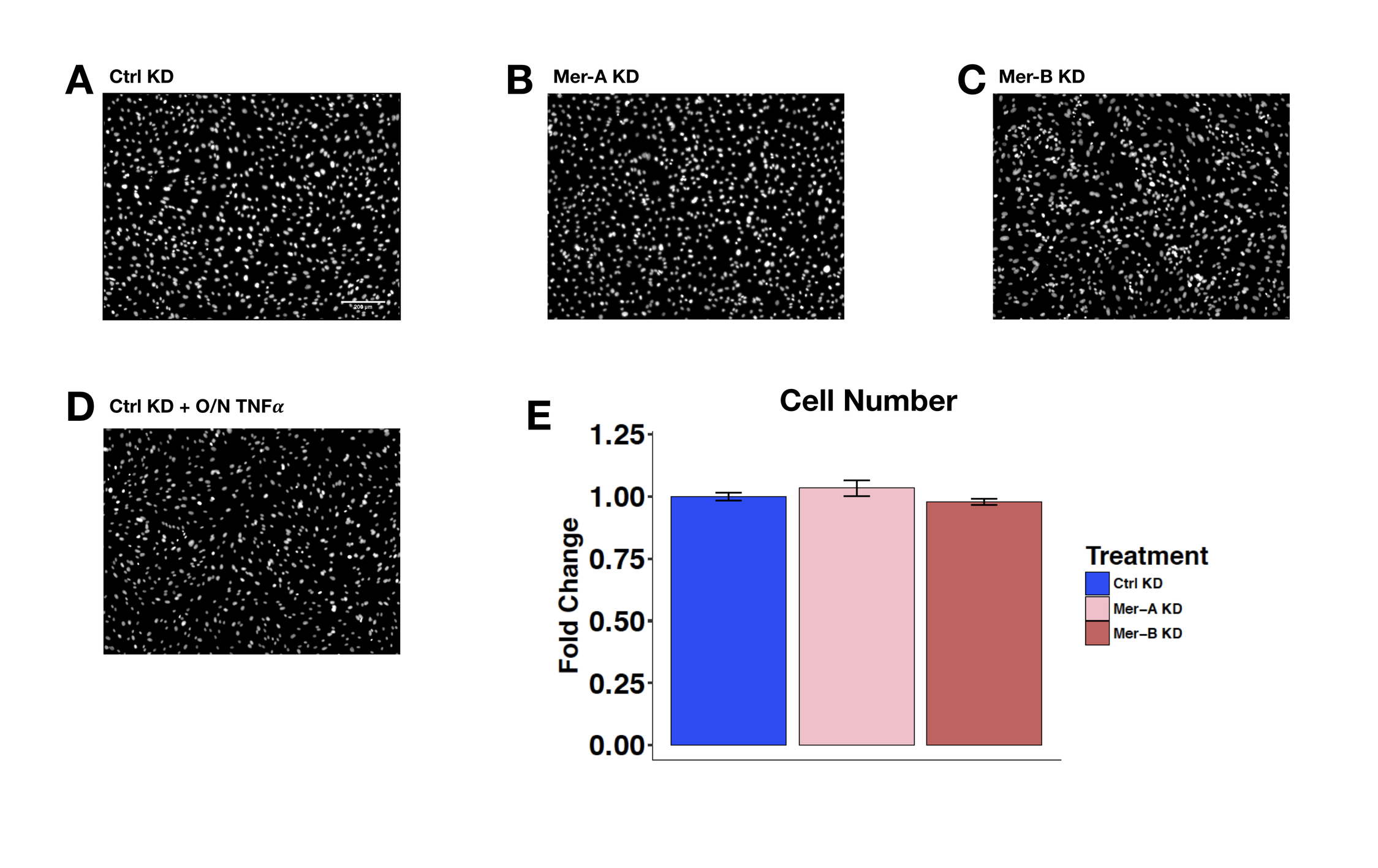

Supplement: S2 Fig — A-D, Representative image fields from XPerT assay, showing cell nuclei (Hoechst stain) from Ctrl KD (A), two different Mer siRNA oligos: Mer-A KD (B) and Mer-B KD (C) ECs. Ctrl KD with O/N TNFα treatment (D) was used as a positive control for the XPerT assay. Scale bar: 200μm. E, Quantification of the number of nuclei per imaging field normalized to Ctrl KD ECs, expressed as fold change. n = 24 imaging fields pooled from 12 coverslips per condition in 2 independent experiments. One-way ANOVA with post hoc Tukey test was used for statistical analyses. (TIF) [file pone.0225051.s002.tif]

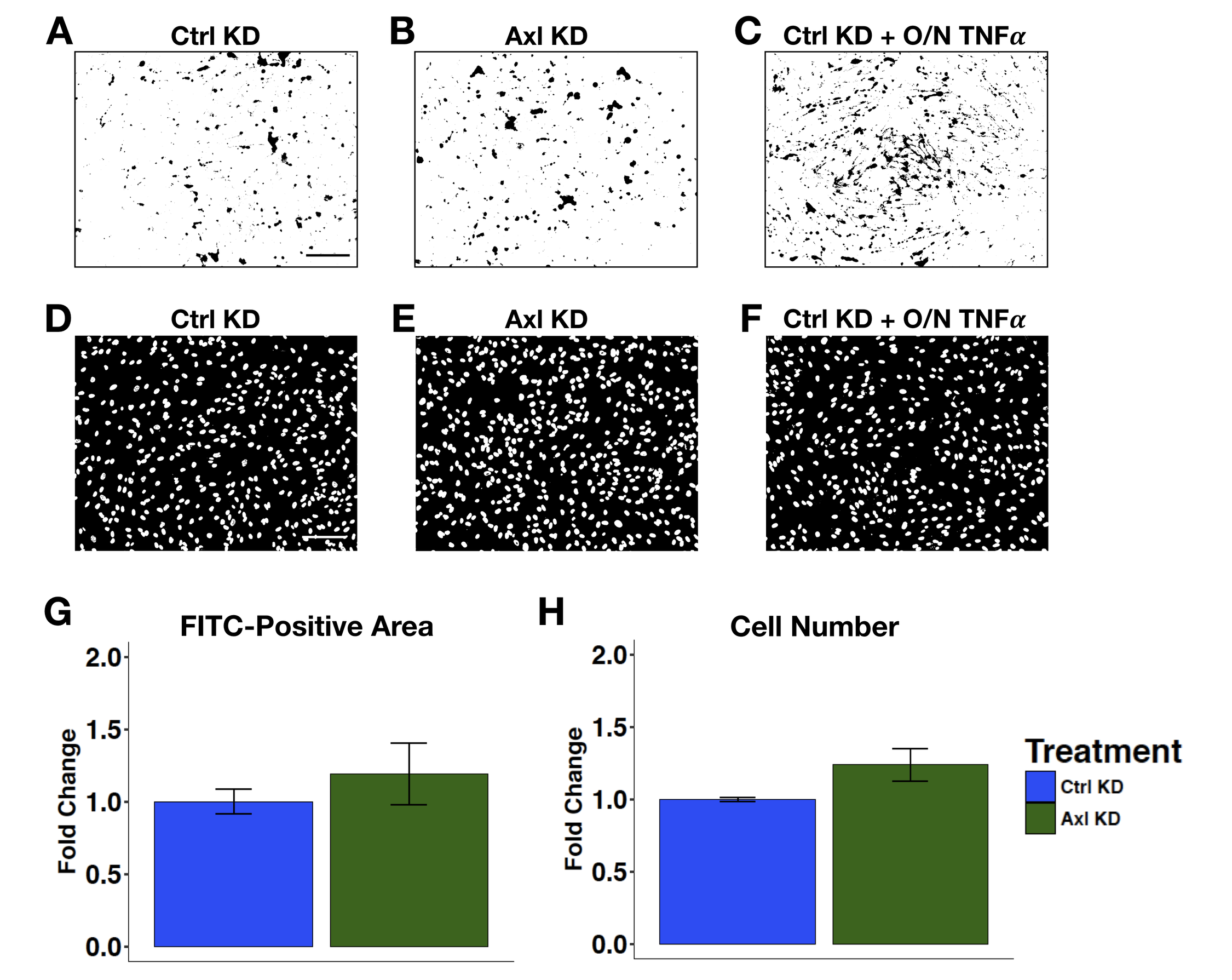

Supplement: S3 Fig — A-C, Permeability test by xPerT assay. Representative thresholded images of local permeability in Ctrl KD (A), and Axl KD (B) EC monolayers, as identified by FITC-streptavidin (black) binding to exposed underlying cell substrate. Ctrl KD with O/N TNF𝜶 treatment (C) was used as a positive control for high permeability. D-F, Representative image fields from XPerT assay, showing EC nuclei (Hoechst stain) from Ctrl KD (D), and Axl KD (E) ECs. Ctrl KD with O/N TNFα treatment (F) was used as a positive control for the XPerT assay. Scale bars in A and D: 200μm. G, Quantification of percent FITC-positive area per imaging field, expressed as fold change normalized to Ctrl KD ECs. H, Quantification of the number of nuclei per imaging field normalized to Ctrl KD ECs, expressed as fold change. n = 12 imaging fields. Results are combined from 4 coverslips per condition in 2 independent experiments. 2-sample student T test was used for statistical analyses. (TIF) [file pone.0225051.s003.tif]

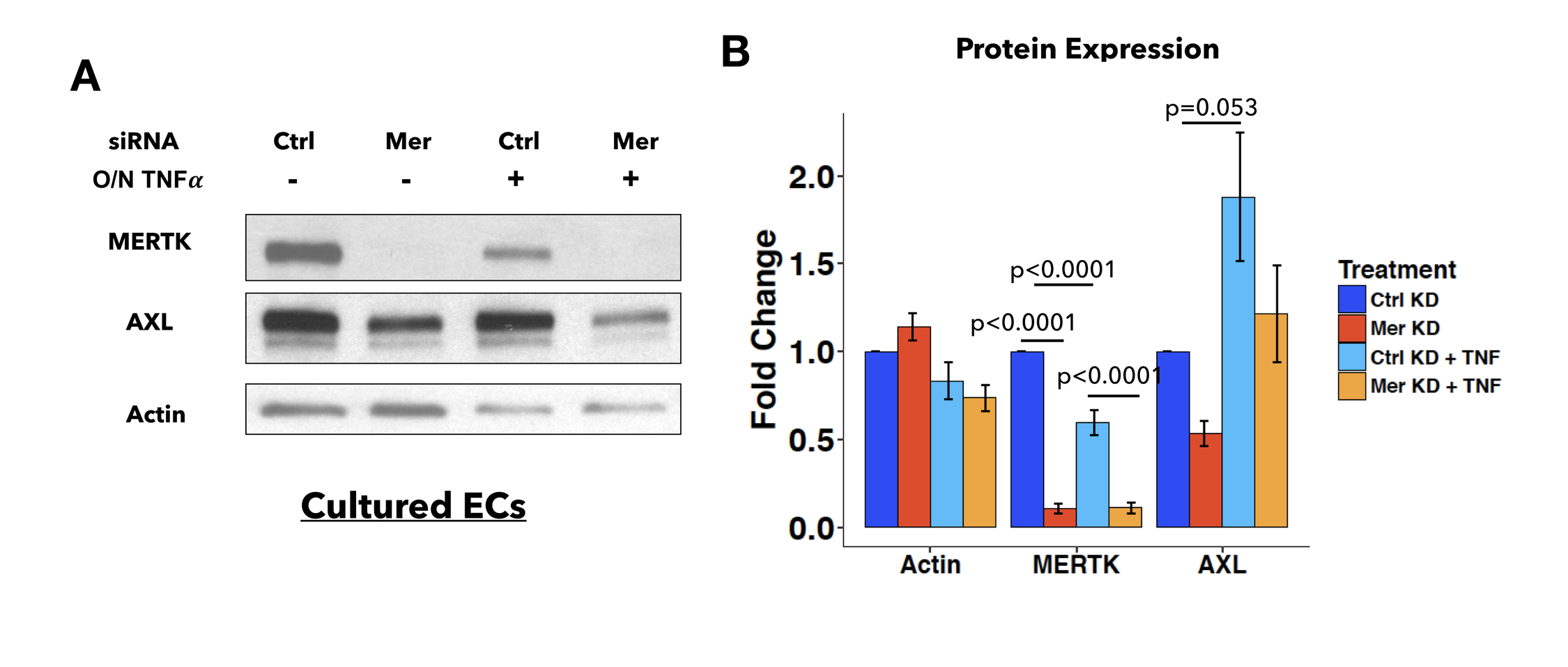

Supplement: S4 Fig — A, Representative immunoblots of MERTK, AXL, and actin in whole cell lysates. ECs pretreated with indicated siRNA oligos were replated at confluent density and cultured in normal medium or TNFα-containing medium overnight before being lysed for immunoblotting. B, Densitometric quantification of protein expression level of actin, MERTK, and AXL. Graph represents fold change normalized to values from Ctrl KD EC in each experiment (n = 8 independent experiments). One-way ANOVA with post hoc Tukey test was used for statistical analyses. (TIF) [file pone.0225051.s004.tif]

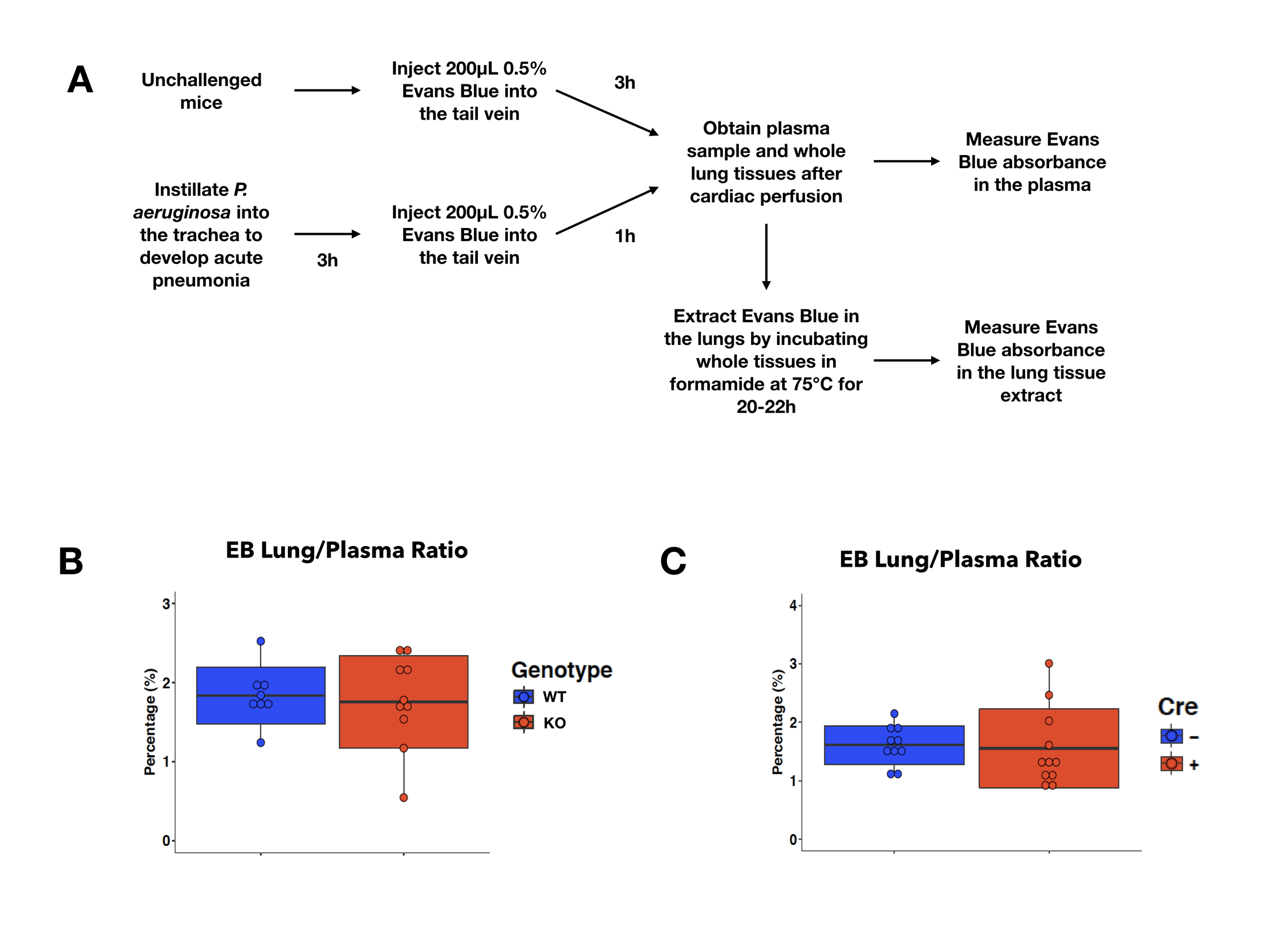

Supplement: S5 Fig — A, Schematic diagram of the Evans blue assay. B, Quantification of Evans blue (EB) leakage into the lungs as expressed by the ratio of EB absorbance measured in whole lung tissues over EB absorbance measured in the plasma from unchallenged WT and KO mice at 3h after EB injection (n = 8 for WT, n = 10 for KO; data pooled from two independent experiments). C, Quantification of EB leakage into the lungs as expressed by the ratio of EB absorbance measured in whole lung tissues over EB absorbance measured in the plasma from unchallenged Cre- and Cre+ mice (n = 10 Cre-; n = 11 Cre+; data pooled from two independent experiments). Two-tail student T test was used for statistical analyses. (TIF) [file pone.0225051.s005.tif]

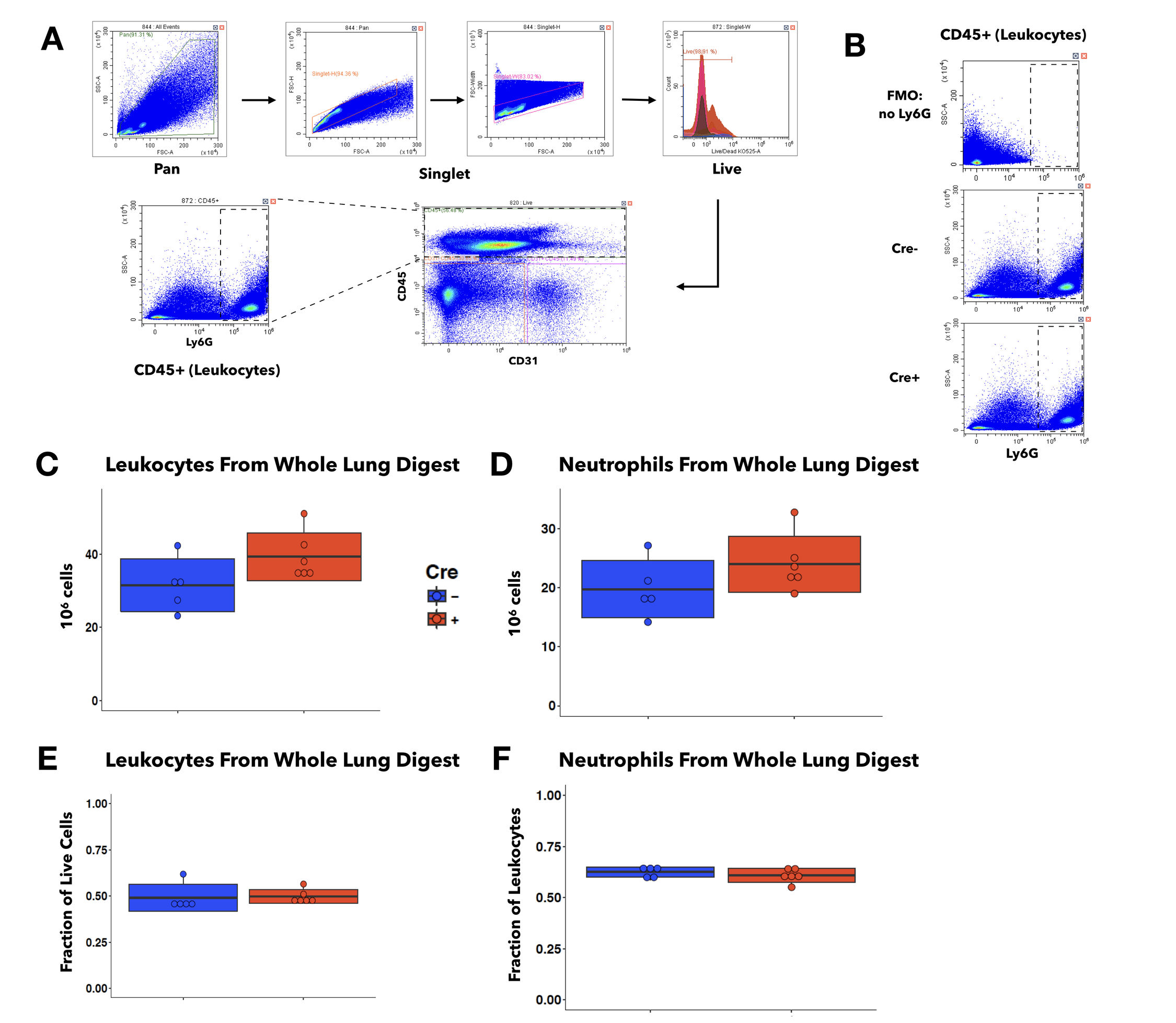

Supplement: S6 Fig — A, Representative images and gating strategies of flow cytometry analyses to isolate leukocyte population (CD45+) from whole lung digest. After singlet cells were identified, dead cells were excluded. By gating on CD45, we identified the CD45+ population as the leukocyte population. The expression of surface Ly6G was then assessed on leukocytes. B, Representative images of Ly6G staining in the CD45+ population. Panels (top to bottom) show cells from fluorescence minus one control (FMO: no Ly6G), Cre-, and Cre+ mice. C-D, Total cell counts of infiltrated leukocytes as identified by CD45+ staining (C), and neutrophils as identified by CD45+ Ly6G+ staining (D) from whole lung digest in Cre- and Cre+ mice. E, Fraction of leukocytes (to live cells) and F, neutrophils (to leukocytes) from whole lung digest in Cre- and Cre+ mice. n = 5 Cre-; n = 6 Cre+ mice from one experiment. Two-tail student T test was used for statistical analyses. (TIF) [file pone.0225051.s006.tif]
