# Supplementary material for: The role of endothelial MERTK during the inflammatory response in lungs
Source: PLoS One. 2019 Dec 5;14(12):e0225051. doi: 10.1371/journal.pone.0225051 (PMC6894824; doi:10.1371/journal.pone.0225051)
Supplement: S1 Raw Images — (PDF) [file pone.0225051.s007.pdf]

# Raw Images of Western Blots

# S1_Raw_Images

**Methods used to generate the blot image**

- All blots were developed with films and scanned at 1200 dpi (color).
- The images were then converted to 8-bit in ImageJ.
- Images of interest were minimally adjusted to ensure best presentation quality and cropped to make figures.
- Moleculare weight markers were labeled in red and blue arrows point to the lanes that were used in the figures in this manuscript.

# Original blots for Figure 1B

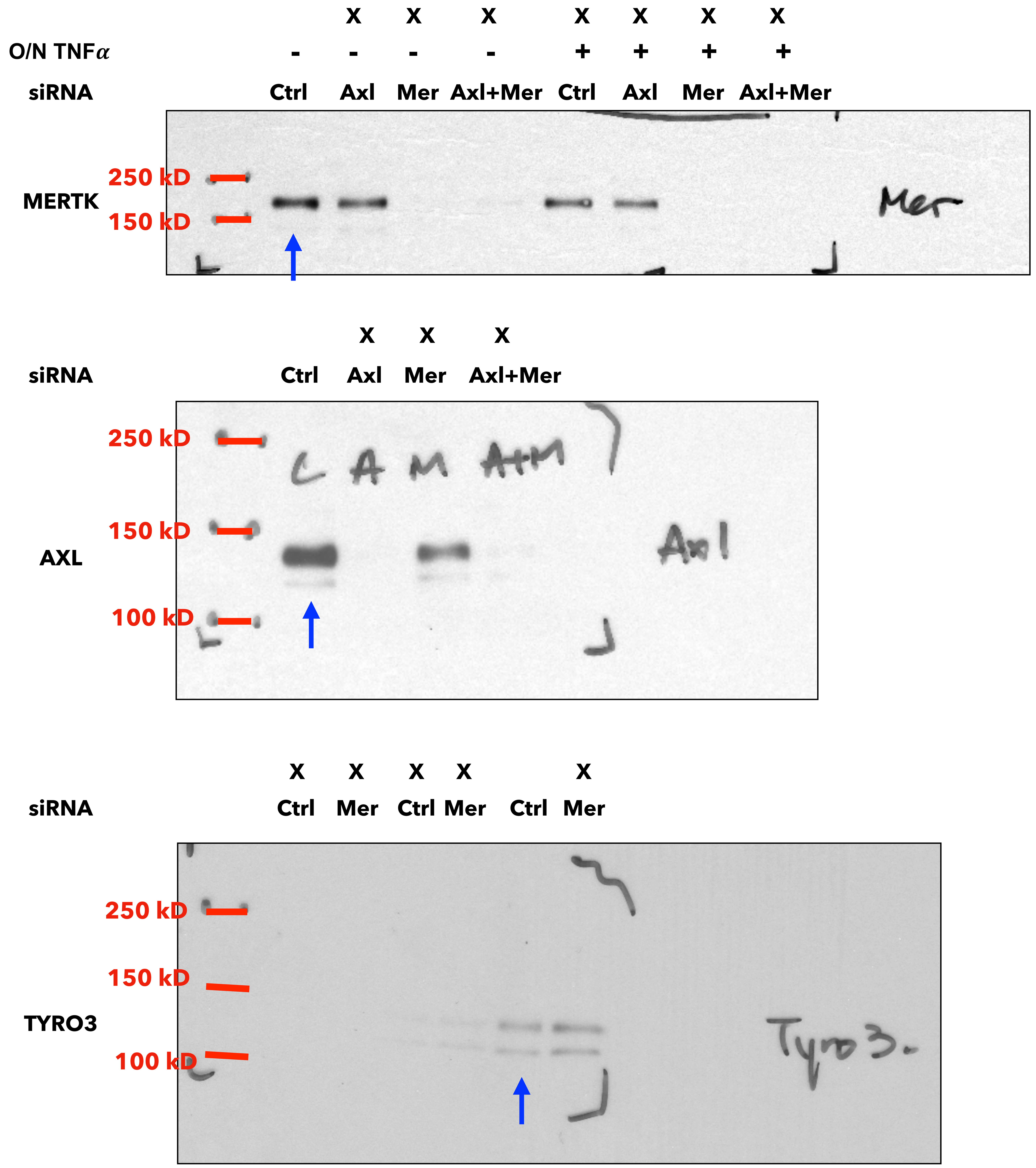


**Cultured ECs**

# Original blots for Figure 2A

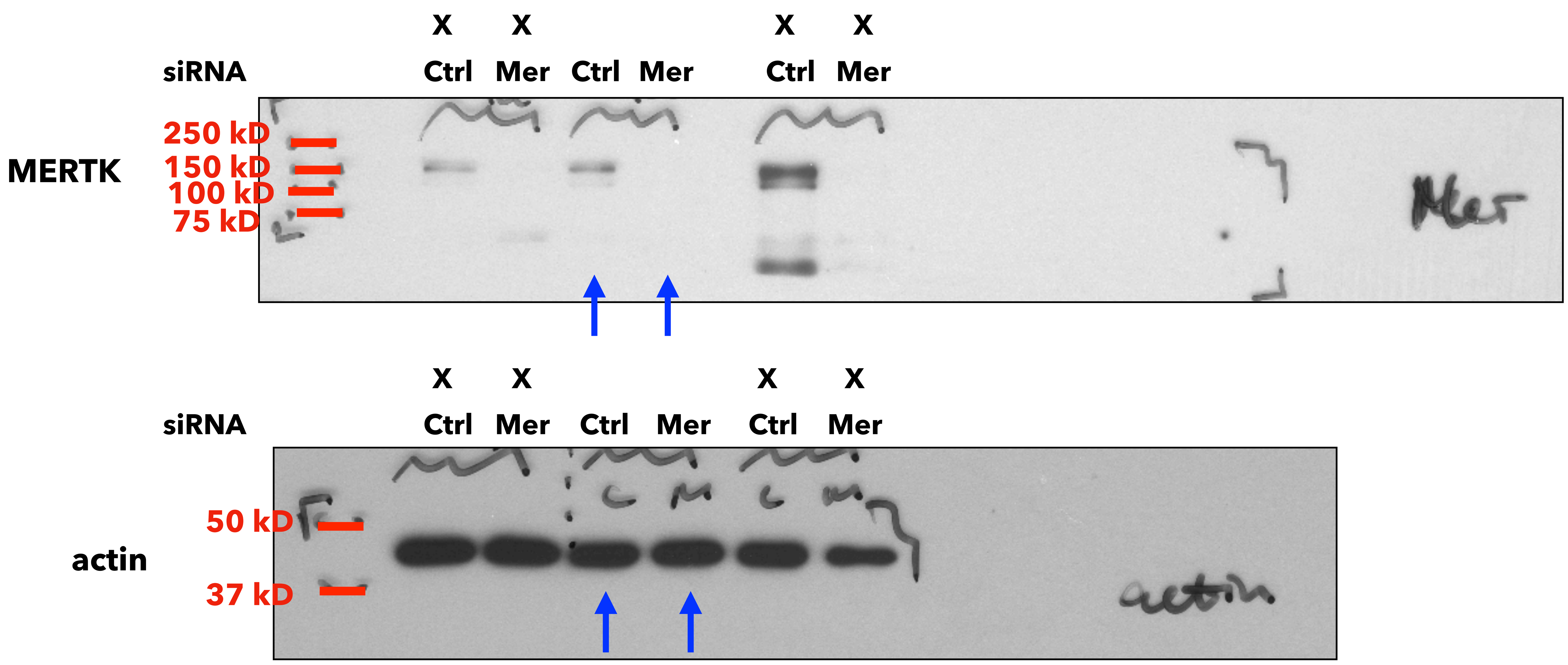


## Cultured ECs

# Original blots for Figure 2F

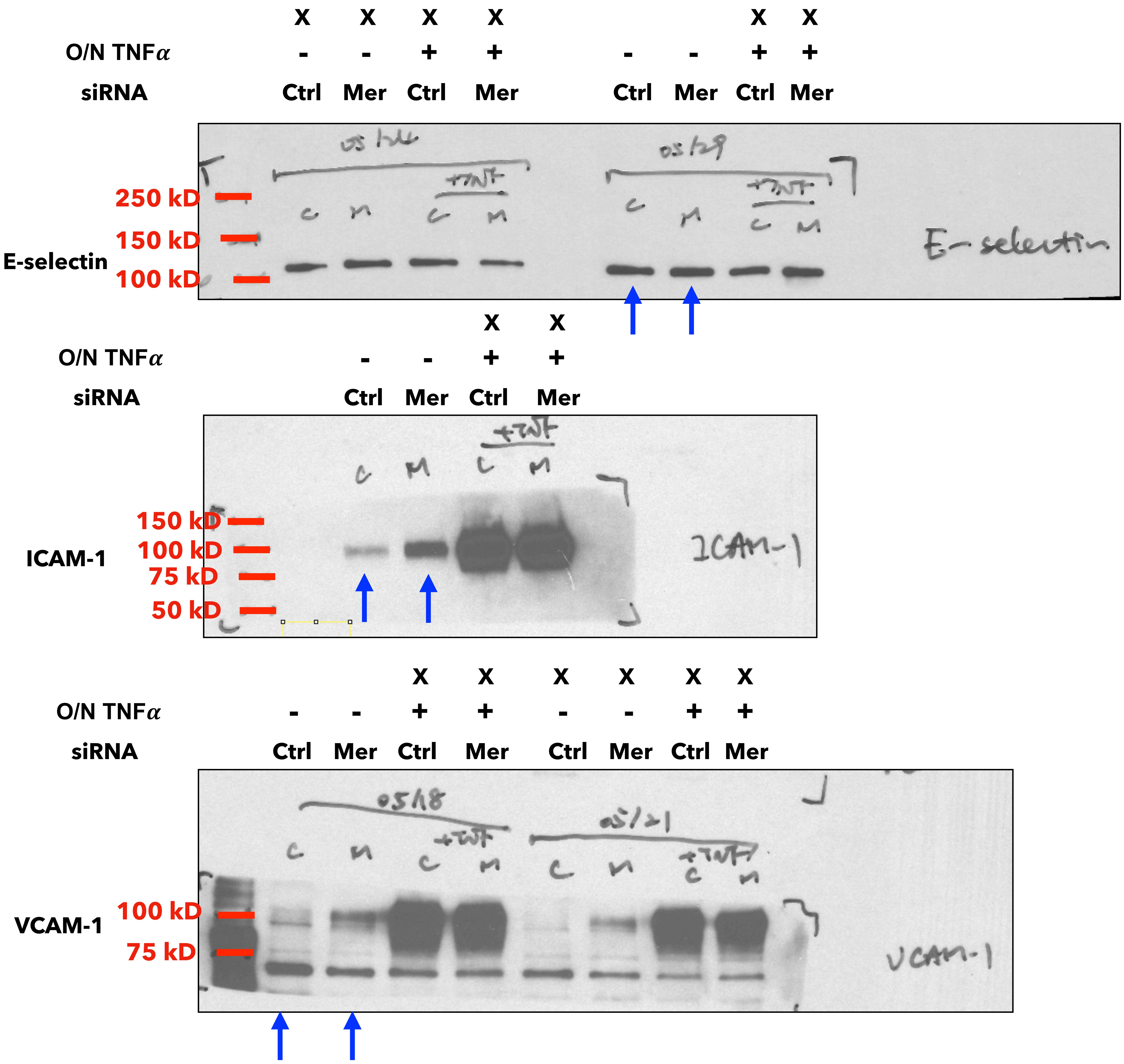


**Cultured ECs**

# Original blots for Figure 2F-continued

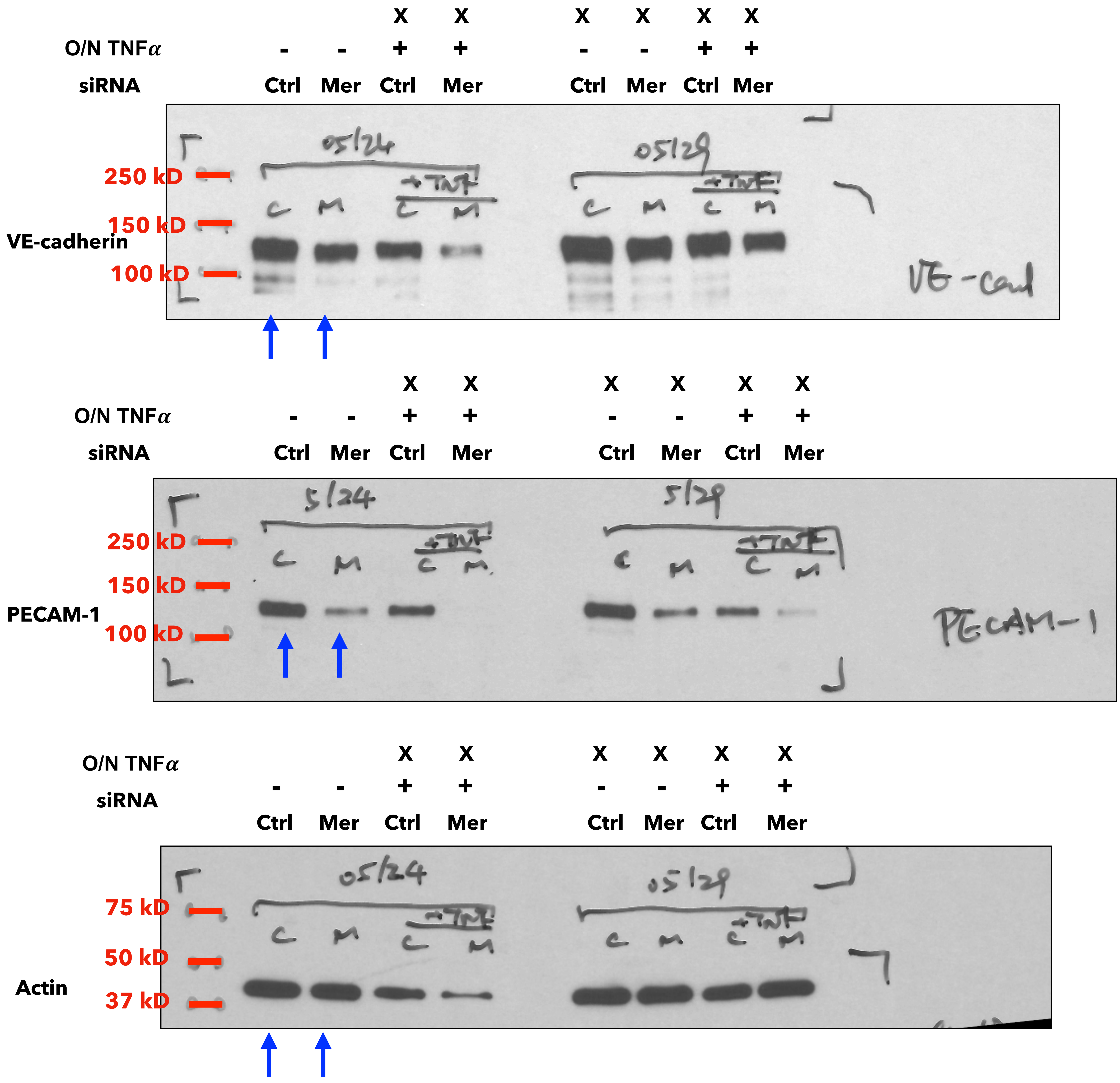


**Cultured ECs**

# Original blots for Figure 5A

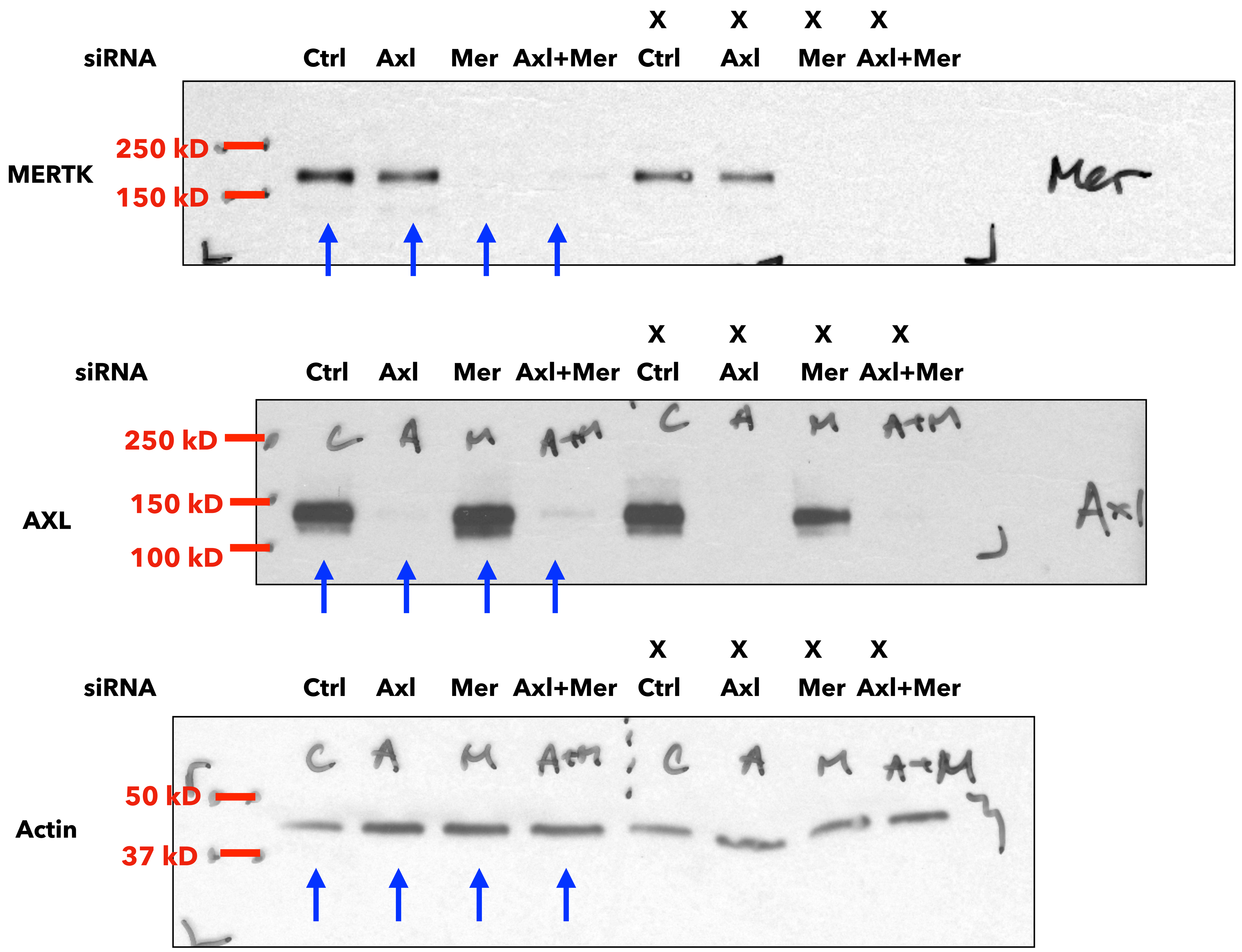


**Cultured ECs**

# Original blots for Figure 6A

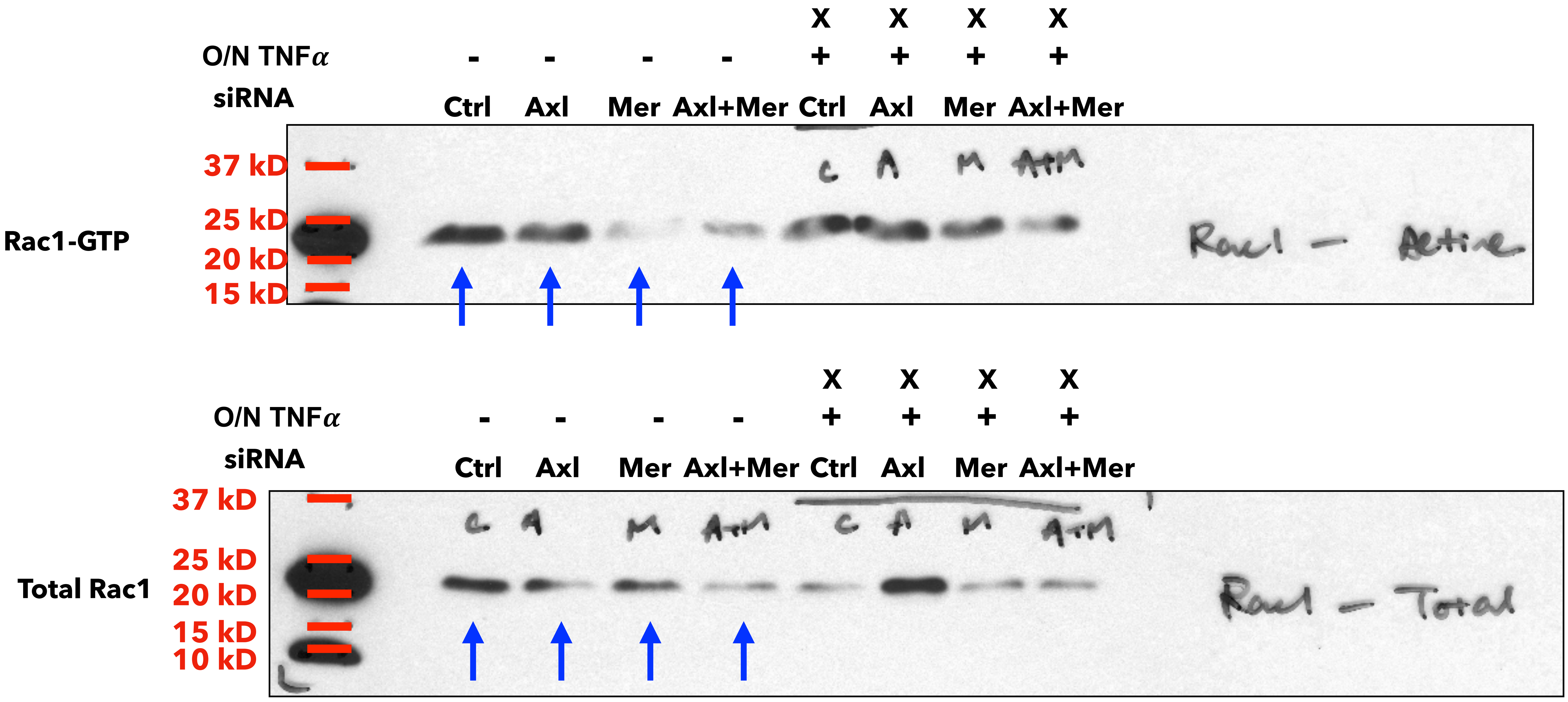


**Cultured ECs**

# Original blots for Figure 6C

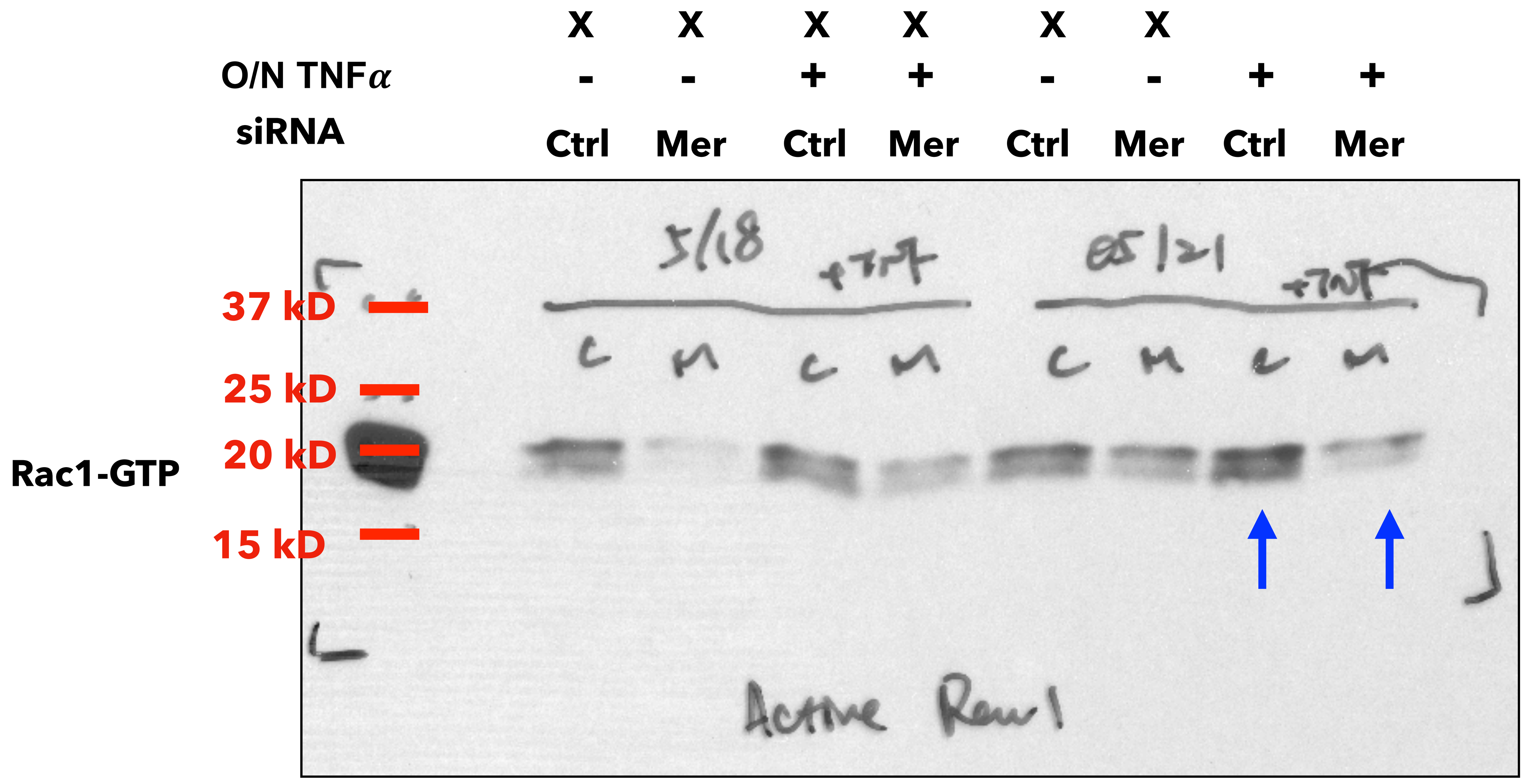


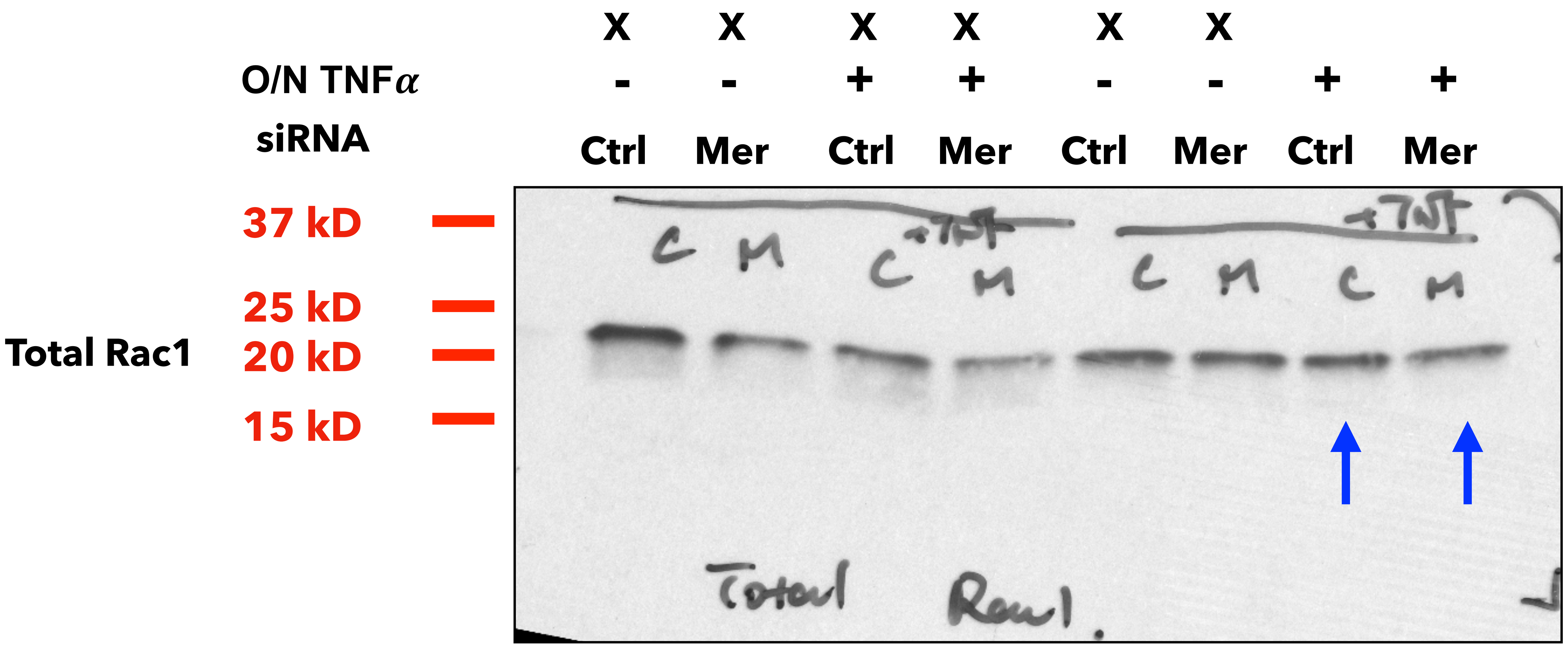


**<u>Cultured ECs</u>**

# Original blots for Figure S1B

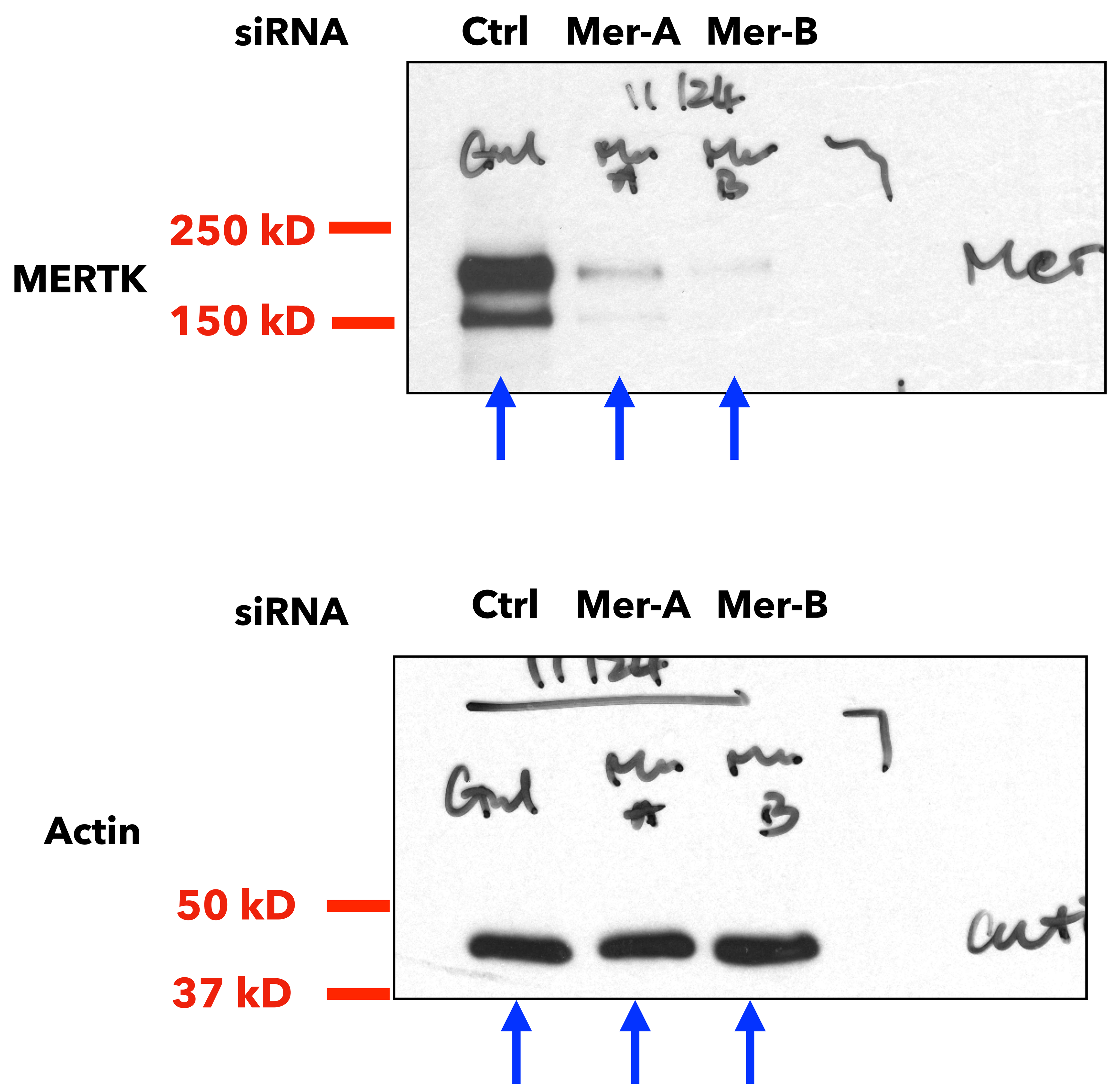


**Cultured ECs**

# Original blots for Figure S4A

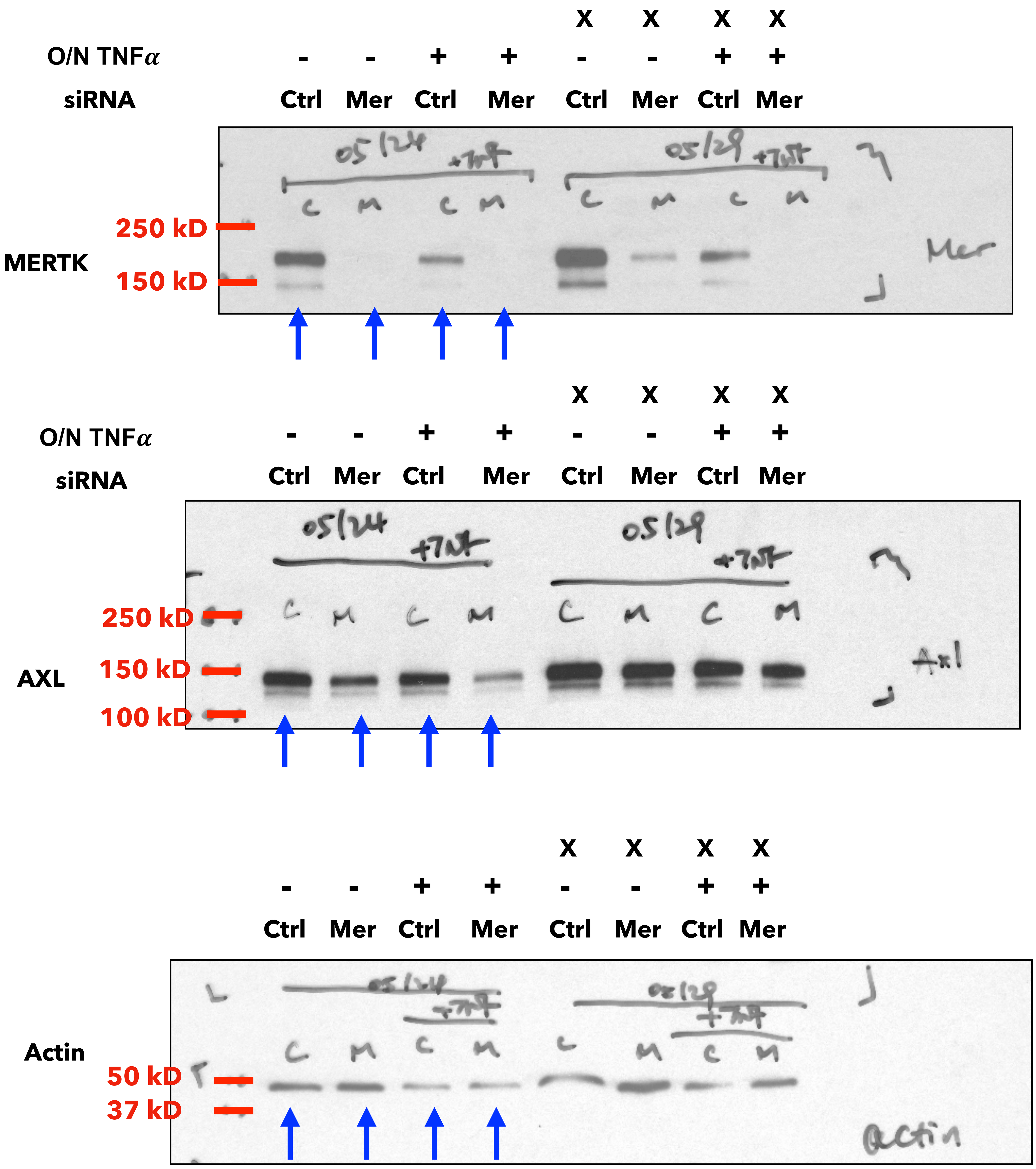


## Cultured ECs
